# The Importance of Reference Materials and Method Validation for Advancing Research on the Health Effects of Dietary Supplements and Other Natural Products

**DOI:** 10.3389/fnut.2021.786261

**Published:** 2021-12-14

**Authors:** Sanem Hosbas Coskun, Stephen A. Wise, Adam J. Kuszak

**Affiliations:** Office of Dietary Supplements, National Institutes of Health, Bethesda, MD, United States

**Keywords:** dietary supplement (DS) analysis, reference material (RM), method validation, chemical characterization, natural product (NP)

## Abstract

Insufficient assessment of the identity and chemical composition of complex natural products, including botanicals, herbal remedies, and dietary supplements, hinders reproducible research and limits understanding mechanism(s) of action and health outcomes, which in turn impede improvements in clinical practice and advances in public health. This review describes available analytical resources and good methodological practices that support natural product characterization and strengthen the knowledge gained for designing and interpreting safety and efficacy investigations. The practice of validating analytical methods demonstrates that measurements of constituents of interest are reproducible and appropriate for the sample (e.g., plant material, phytochemical extract, and biological specimen). In particular, the utilization of matrix-based reference materials enables researchers to assess the accuracy, precision, and sensitivity of analytical measurements of natural product constituents, including dietary ingredients and their metabolites. Select case studies are presented where the careful application of these resources and practices has enhanced experimental rigor and benefited research on dietary supplement health effects.

## Introduction

Dietary supplements (DS) and other natural products (NPs) are repeatedly investigated in cellular systems, observational studies, and randomized controlled trials due to their prevalent use for potential health benefits (1–3). However, outcomes from clinical studies on botanical-derived NP efficacy for health maintenance and disease risk reduction often yield mixed results, driven in part by the varying composition of the experimental interventions investigated (4). Clinical trials studying the effect of *Echinacea* species in respiratory infections, for example, investigate varying doses and different species and plant parts (5–7). Since the early 2000's, seminal papers have described the need for sufficient reporting in clinical research on botanicals, dietary supplements, and traditional medicines, including elaborations on the Consolidated Standards of Reporting Trials (CONSORT) guidelines (8–11). However, even though recent studies have found an overall improved reporting quality in studies of certain NPs, there is still an indication that insufficient reporting details on methodology and characterization continue to be an issue. For example, an assessment of randomized trials of Asian ginseng *(Panax ginseng* C.A. Meyer) and North American ginseng (*Panax quinquefolius* L.) deemed that <40% of trials conducted from 1980 to 2019 adequately addressed CONSORT criteria for methodology reporting, and <15% provided sufficient details on intervention composition to allow for experimental replication (12). Such insufficient characterization of an investigational DS or NP chemical composition reduces the capability for data analysis, limits research reproducibility, and impedes the continuity of scientific progress (4, 13, 14).

Analytical characterization of DS and NP interventions is essential for rigorous basic and clinical research on their health effects. Detailed chemical characterization improves research reproducibility, as investigators' ability to replicate and build upon studies is substantially increased the more that is known about an intervention's composition. Sufficient characterization also facilitates more meaningful comparisons of experimental design and data interpretation across studies, as it can be difficult, if not impossible, to interpret the public health relevance of a study on NP efficacy using a preparation with poorly understood composition (15). The insight gained from investigations of a NP's mechanism(s) of action, pharmacokinetics, or herb/drug interactions are significantly expanded through a better understanding of its chemical composition. In addition to better understanding the basis for positive health effects, NP chemical characterization is necessary for adequate safety assessment, a prerequisite for any clinical investigation, and particularly important for botanical-derived interventions where adulteration of source materials is a known issue (16, 17). This review describes the importance of utilizing reference materials and validated methods to address these analytical challenges and enhance natural product research rigor and reproducibility. A discussion of important considerations in method validation, an assessment of available certified reference materials, and case studies provide guidance and good practices for advancing research on the health effects of DS and other NPs. Comprehensive tables index examples where reference material use has facilitated innovative method development and/or supported novel research.

## Key Considerations for DS and NP Characterization

The major characterization parameters for complex NPs such as DS include confirmation of identity/authenticity, quantification of known or putative bioactive constituents or marker compounds, an assessment of purity/composition, and safety evaluation (4, 18). For certain vitamins and minerals, it is important for investigators to consider whether the isoforms or chelation states are appropriate to the research hypothesis and experimental design. For example, vitamin E comprises a group of eight chemical isoforms that have varying biological activities, while chelates of trace minerals may alter bioavailability. For botanicals, it is essential to verify plant species identity and authenticity, confirm the correct plant parts were used, and test for the presence of harmful compounds, microbes, pesticides, toxins, and toxic elements (e.g., cadmium, mercury, lead, and arsenic). Batch-to-batch reproducibility and stability of experimental NP interventions should also be confirmed. Investigators should assess these key characterization parameters to the extent practicable to best support advances in NP biomedical research.

Well-designed research must control as many variables as possible to support replicable results. Exploratory and molecular/cellular studies should characterize NP interventions to the extent feasible to increase the potential for gaining insight into underlying biological mechanisms. Animal models and clinical studies of DS and other NPs should utilize test materials that have been standardized to the extent possible. For botanical-derived DS, standardization starts with species verification and continues through chemical identification and quantification of specific compounds which are known or hypothesized to produce a biological activity. Often the particular compound or compounds responsible for an activity are not known, and marker compounds must instead be chosen for standardization. The interpretation of pre-clinical investigations of metabolic pathways and safety, critical to assess before moving to translational research in human subjects, also rests on accurate compositional characterization (19).

Throughout all these considerations, accurate, precise, and reliable analytical measurements are needed to assess the consistency and quality of NPs obtained from various suppliers, confirm the repeatability of product preparation schemes, and assure safety by detecting toxic constituents. DS products are prepared in numerous formulations, including powders, liquids, tablets, capsules, and chewable gels (“gummies”), which adds unique analytical challenges to homogenization, extraction, and reproducible quantification. A limited number of contract research organizations and independent laboratories have the capability and experience to analyze DS (20). Therefore, it is incumbent upon biomedical researchers to assess in-house controls and demonstrate the accuracy of their analytical methods.

## Benefits of Method Validation and RMs in NP Biomedical Research

The requisite level of characterization and standardization for DS and other NP research is facilitated by using validated analytical methods and matrix-matched reference materials for accurate quantification of nutrients, minerals, phytochemicals, metabolites, and toxic analytes. Analytical methods employed in NP authentication and characterization must be carefully selected and controlled to ensure the accuracy and precision of quantitative measurements for phytochemicals, nutrients, and possible contaminants (21). Methods should be fit for purpose, meaning the measurements are sufficiently reliable and appropriate for the sample matrix (e.g., ground plant part, liquid extract, and capsule formulation). Formal validation studies of analytical methods are the means to demonstrate fitness for purpose and reliability through the determination of measurement performance parameters, including precision, accuracy, selectivity, specificity, limit of detection, limit of quantitation, and reproducibility. Therefore, utilizing a validated method is an optimal approach for demonstrating unbiased and reliable measurements, transferring projects or analyses to new lab members, and comparing research results across multiple labs.

Through the processes of method development and validation, it is paramount that researchers confirm their methods are generating the correct answers for analyte quantification or material authentication, and this is where reference materials play a vital role. The use of reference materials to assure the quality of analytical measurements is well-defined in analytical chemistry, particularly in environmental, clinical, and food analyses. A review by Ulberth summarizes the international terminology for the types of reference materials and their use (22). A reference material (RM) is a “material, sufficiently homogeneous and stable for one or more specified properties, which has been established to be fit for its intended use in a measurement process.”^1^ A certified reference material (CRM) is defined as a “RM characterized by a metrologically valid procedure for one or more specified properties, accompanied by an RM certificate that provides the value of the specified property, its associated uncertainty, and a statement of metrological traceability” (see text footnote 1). For example, a St. John's Wort (*Hypericum perforatum* L.) CRM could comprise a homogenized powder prepared from authenticated aerial parts, with quantified values for hypericin. CRMs of known quantities of analytes in solution are intended for use as calibration solutions, as described later.

The inherent complexity of natural product preparations and the resulting analytical challenges, such as accounting for extraction efficiency and interfering compounds, are best addressed by matrix-based reference materials. Compared to the myriad NPs and botanicals used worldwide there is a comparatively small number of matrix based RMs available. However, it is important for analysts and researchers to consider that RMs are often not intended to be representative of every possible matrix, nor are they intended to represent a “gold standard” for an ingredient or formulated product. Rather, RMs are meant to be representative of the analytical challenges encountered with similar matrices, e.g., isoflavone extraction from a leaf material. Depending on the purpose of the RM and the analytical question being asked, an exact matrix-matched RM is not necessarily required for method development and validation or NP characterization in research. As such, the limited number of currently available matrix based RMs can be applicable to the characterization of a much larger number of matrices, and they should be used wherever possible when quantification of marker compounds and/or toxic metal contaminants is required. CRMs can be used as quality control (QC) materials to determine bioactive/marker compound content, detect contamination, or assign values to verify in-house QC materials. Researchers using botanical supplements in clinical studies can, and wherever possible should, verify the accuracy of any chemical characterization of the experimental intervention by using CRMs as QC materials.

## Good Practices in Analytical Method Validation

Standard-setting organizations and regulatory agencies have provided detailed guidance and workflow schema on how to conduct formal validation studies of analytical methods specifically for NPs and dietary ingredients^2^^,^^3^^,^^4^. These validation guidance documents define parameters that should be assessed for qualitative or quantitative methods and outline procedures for establishing a measurement's linearity range and reliability. While there is not a consensus agreement of which analytical parameters must be assessed to constitute a formal validation, there are commonalities across the guidelines of different organizations. A quantitative method's selectivity and specificity, accuracy, precision, recovery, limit of detection, limit of quantification, repeatability, and reproducibility are key parameters that should be assessed in a formal validation. A qualitative method, for example one that is intended for botanical identity or authenticity, should also assess specificity, selectivity, and limit of detection, as well as false positive/negative rates. Validation guidelines specific to identification methods also describe a statistical modeling procedure, termed the probability of identification, as a key parameter to assess a qualitative method's reliability (23).

Validated methods for DS ingredients and products can be leveraged in both industry and academic settings to enhance measurement confidence and reproducibility. Biomedical researchers focused on delineating the connections between dietary ingredients, their metabolism, mechanisms of action, and health outcomes have used validation to establish the accuracy and reproducibility of their measurements. As examples, published validation studies of a liquid chromatography—mass spectrometry (LC-MS) quantification of soy sphingadienes under investigation for chemopreventive activity (24), a gas chromatography (GC)-MS characterization of a complex grape seed flavanol mixture studied in models of stress resilience (25), a high performance liquid chromatography (HPLC)-tandem mass spectrometry (MS/MS) determination of cholecalciferol in clinical serum and plasma samples (26), and an ultra-high performance liquid chromatography (UHPLC)-MS/MS quantification of red clover isoflavones and milk thistle flavonolignans in supplements and human serum (27) all contribute significantly to experimental rigor in subsequent hypothesis-driven investigations and help build confidence in translational NP research.

## Good Practices in the Use of Matrix Reference Materials

There are three types of CRMs with different complexity and uses (22, 28): (1) pure substance (neat chemical), (2) calibration solution/mixture containing one or more constituents, and (3) natural matrix materials. Pure substance CRMs and solution CRMs are closely related in that the pure substances are generally intended as primary standards (of known purity) for use in preparing calibration solutions, and solution CRMs are typically intended for use directly as a calibration solution, i.e., eliminating the step of the analyst preparing a solution from a pure substance. These solution CRMs are typically used to calibrate analytical instruments and, in the case where chromatographic separations are involved, confirm retention times and determine detector response for the analytes of interest. Natural matrix CRMs, which ideally should be compositionally similar to the real-world samples analyzed, are used to evaluate the complete analytical measurement process, including dissolution or solvent extraction of the matrix, clean-up of the extract, isolation and/or enrichment of the constituents of interest, and the final instrumental analysis including chromatographic separation, detection, and quantification. Matrix RMs play a critical role in validating the complete analytical method and assessing the accuracy and comparability of results among different laboratories over time.

**Figure 1:**
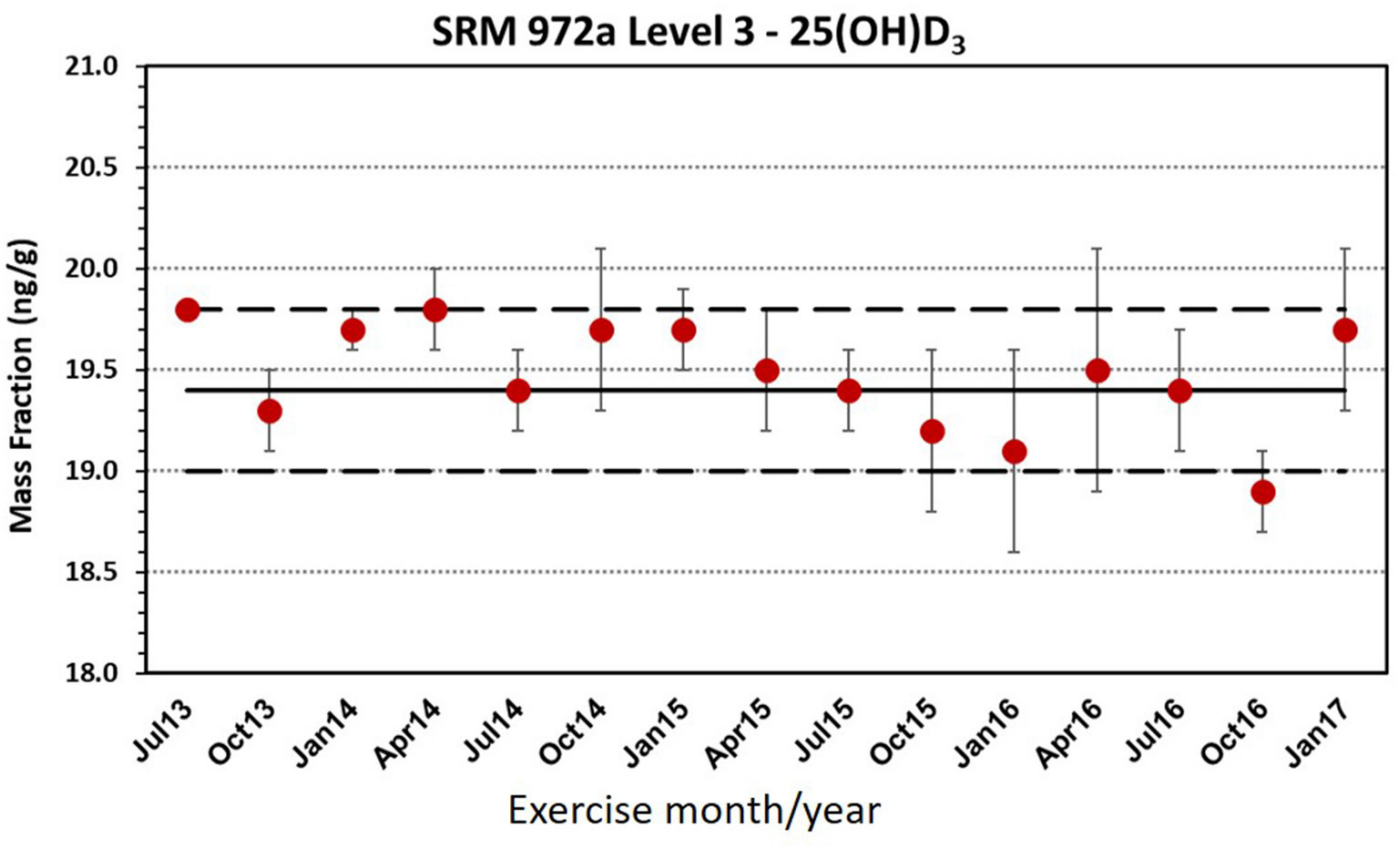
Control chart for the NIST determination of 25(OH)D_3_ in SRM 972a for DEQAS exercises from July 2013 to January 2017. Error bars are ± SD for duplicate analyses of SRM 972a. Solid line is the certified value and dashed line is the uncertainty of the certified value. Burdette et al. (35), adapted by permission of AOAC International (aoac.org).

Matrix CRMs are intended for the following applications: (1) analytical method development and method validation, i.e., to assess accuracy or trueness of measurement results, (2) to serve as QC materials, (3) to assign values for in-house QC materials, and (4) to provide metrological traceability of measurement results. Practical guides to the use of CRMs are provided by several European Reference Materials Application Notes (29, 30) and by Sharpless et al. (31). Matrix CRMs should not, however, be used for the calibration of analytical instruments. The use of matrix CRMs that are similar in matrix to the actual samples analyzed is critical in new method development and validation for assessing the accuracy of the complete analytical measurement process, e.g., extraction and dissolution, clean-up, and finally, chromatographic separation and detection. Several excellent studies from the environmental measurement area have been reported that illustrate the value of using a CRM to evaluate the efficacy of solvent extraction techniques (32–34). CRMs can be used as QC materials during routine measurements through their inclusion in each batch of actual samples to assess the accuracy or trueness of the results and by preparing control charts to monitor the quality of the measurements over time. An example of using a CRM in a control chart is illustrated in Figure 1, where a CRM for 25-hydroxyvitamin D_3_ in human serum was analyzed quarterly over a period of 5 years as part of an international external quality assurance program, i.e., Vitamin D External Quality Assessment Scheme (DEQAS) (35). Due to the cost of CRMs, laboratories may prepare in-house QC materials and analyze the CRM with the in-house QC materials to assign values (i.e., traceability) and then use the in-house QC material for routine measurements (31).

Finally, CRMs have proven useful in novel, exploratory and hypothesis-driven research because they are homogeneous and stable materials that are widely available to the research community, characteristics which also facilitate collaboration and comparison of results. For instance, if researchers characterize different ginseng materials as part of a study to identify new marker compounds (i.e., not the constituents with values assigned by the CRM producer), ginseng CRMs can be included as part of the study and the published results for the content of these novel marker compounds in the CRM can be referenced and compared to other laboratory analyses in the future. An excellent example of researchers analyzing CRMs for the determination of new analytes is work by Zhu and Hites, where they used a newly-developed method for the determination of the emerging environmental contaminant polybrominated diphenyl ethers (PBDEs) in several existing National Institute of Standards and Technology (NIST) marine and freshwater tissue matrix CRMs (36). Zhu and Hites published the first report of concentrations of PBDEs in these CRMs and stated, “Given the availability and homogeneity of the NIST SRMs, we suggest that these materials can be used for the interlaboratory calibration of PBDE concentrations” (36). Years later, NIST assigned certified values for selected PBDE congeners in these SRMs (37), further increasing their analytical utility.

## Current Availability of Reference Materials for Dietary Supplements

Within the dietary supplement industry, the term reference material typically refers to “authentic reference standards” for compounds and/or ingredients found in supplement products. In the early 2000's a limited number of reference standards for botanicals or other DS ingredients were commercially available. However, they typically had limited information on the purity of the material, and quantitative information on the chemical content of natural matrix RMs for botanical dietary supplement ingredients was intended for testing against a limited number of compendial standards (38). In 2002 the U.S. National Institutes of Health Office of Dietary Supplements (NIH-ODS) established the Analytical Methods and Reference Materials (AMRM) Program,^5^ with a mission to enhance rigorous dietary supplement research support and quantitative analysis (39). To meet industry and research needs for quantitative reference materials that were not tied to compendial testing, NIH-ODS collaborated with NIST to develop CRMs for dietary ingredients and supplement products.^6^ CRMs issued by NIST are called Standard Reference Materials (SRMs), the majority of which are characterized for chemical composition. NIST is the national metrology institute (NMI) within the U.S.; while most matrix CRMs for chemical content are produced by NMIs in their respective countries, they are often distributed worldwide. Commercial suppliers, such as Cerilliant/MilliporeSigma,^7^ may also produce CRMs and RMs for certain constituents of dietary supplements.

In addition to utilizing CRMs, which are the major focus of this review, DS and NP researchers can also take advantage of available standards from multiple sources to aid in botanical identification and authentication. The United States Pharmacopeia (USP) develops pure chemical and matrix-based reference standards for use in conjunction with their formal documentary standards (monographs) for the characterization and quality assessment of therapeutics, foods, and DS. These USP analytical tools are intended to aid the verification of identity and composition of ingredients used in drug formulations and DS products, and their catalog of reference standards includes vitamins, minerals, phytochemicals, and complex botanical preparations (40, 41). The American Herbal Pharmacopeia produces qualitative monographs and matching authenticated reference standards with a focus on botanicals and herbal medicines.^8^ Some commercial reagent suppliers, such as ChromaDex,^9^ PhytoLab,^10^ Alkemist,^11^ and Extrasynthese^12^ also produce nutrient, phytochemical and/or plant material reference standards which can be used to support method development and chemical characterizations of raw materials or experimental interventions. While these types of reference standards and vouchered specimens may not offer the same level of quantitative values for specified bioactive constituents or other properties, their use can play a significant role in providing a well-characterized control material that can readily be used to benefit a research collaboration, compare results across studies, and support experimental replication.

### NIH-ODS/NIST Supported Development of Reference Materials

Initial efforts of the NIH-ODS/NIST collaboration focused on the development of authentic botanical ingredient-containing SRMs and RMs with values assigned for the content of active and/or marker compounds for use in verification of content and manufacturing quality control, particularly to address safety concerns related to contaminants such as toxic elements (42–44). Within the dietary supplement industry, chemical analyses are typically performed on raw materials (plants and extracts of plants) and finished products (e.g., tablets). Thus, NIST SRMs were designed wherever possible to comprise a “suite” of materials for each botanical dietary supplement ingredient consisting of authentic plant material, an extract of the plant material, and the finished product. The intent was to provide the various matrices encountered in the market and used in research that may yield distinct analytical challenges, e.g., different concentrations of constituents of interest, differences in extractability of constituents from the matrix, and various potential interferences.

Certified reference materials for ephedra was the first high priority of the NIH-ODS/NIST collaboration and a suite of SRMs was developed, including aerial plant parts, extracts, solid oral dosage form (SODF), and ephedra-containing protein powder. Despite the discontinuation of this SRM suite following the U.S. Food and Drug Administration (FDA)'s ban of ephedra, the experience provided the model for the botanical matrix SRMs that have been developed subsequently (45). Botanical dietary supplement matrix CRMs, almost all of which are SRMs developed by NIST in collaboration with NIH-ODS during the past 20 years, are summarized in Table 1. In 2009, the portfolio of dietary supplement SRMs expanded to non-botanical supplements with the development of SRM 3280 Multielement/Multivitamin Tablets (46) as shown in Table 2 and later included tocopherols in oil, chromium-containing SODF, and iodized salt. Several botanical dietary supplement NIST SRMs that are currently in development are listed in Table 3.

**Table 1:** Currently available botanical dietary supplement matrix CRMs.

**Source**	**CRM No**.	**CRM description**	**Certified values**	**Reference values**	**Total values**
NIST	3246	Ginkgo (*Ginkgo biloba*) leaves	Flavonoids (4); ginkgolides (1); toxic elements (3); DNA sequence (identity)	Flavonoids (4); ginkgolides (4); bilobalide (1)	**18**
NIST	3247	Ginkgo (*Ginkgo biloba*) extract	Flavonoids (4); ginkgolides and bilobalide (6); toxic elements (1)	Toxic elements (2)	**13**
NIST	3248	Ginkgo-containing SODF	Flavonoids (4); ginkgolides (3); toxic elements (1)	Ginkgolides (2); bilobalide (1); toxic elements (3)	**14**
NIST	3250	Saw palmetto (*Serenoa repens*) Fruit	Phytosterols (3), fatty acids (14)	Phytosterols (3), fatty acids (4), free fatty acids (16)	**40**
NIST	3251	Saw palmetto (*Serenoa repens*) Extract	Phytosterols (3); fatty acids (17); carotenoids (1); vitamins (1)	Phytosterols (3); fatty acids (3); free fatty acids (17); tocopherol (1); carotenoids (2); cycloartenol	**48**
NIST	3274	Botanical Oils Containing Omega-3 and Omega-6 Fatty Acids	Fatty acids (35)	Fatty acids (33)	**68**
NIST	3274-1	Borage oil (*Borago officinalis*)	Fatty acids (9)	Fatty acids (8)	**17**
NIST	3274-2	Evening Primrose oil *(Oenothera biennis*)	Fatty acids (10)	Fatty acids (8)	**18**
NIST	3274-3	Flax (*Linium usitatissimum*) Seed	Fatty acids (9)	Fatty acids (7)	**16**
NIST	3274-4	Perilla (*Perilla frutescens*)	Fatty acids (7)	Fatty acids (10)	**17**
NIST	3281	Cranberry (*Vaccinium macrocarpon*) Fruit	Organic acids (1); elements (9)	Proximates (5); sugars (3); elements (2); anthocyanidins (3)	**25**
NIST	3282	Low-Calorie Cranberry Juice Cocktail	Organic acids (3); elements (6)	Organic acids (6); anions (2); sugars (3); elements (2)	**22**
NIST	3283	Cranberry (*Vaccinium macrocarpon*) Extract	Organic acids (3)	Organic acids (6); anions (2)	**14**
NIST	3284	Cranberry-Containing SODF	Organic acids (3)	Organic acids (4); anions (2)	**12**
NIST	3285	Mixed Berry-Containing SODF	Organic acids (2)	Organic acids (6); anions (2)	**12**
NIST	3287	Blueberry (*Vaccinium corymbosum*) Fruit	Organic acids (1); vitamins (4); elements (8)	Organic acids (5); proximates (6) and fiber; sugars (3): elements (1); amino acids (16); anions (2)	**50**
NIST	3291	Bilberry extract (*Vaccinium myrtillus*) Extract	Organic acids (3)	Organic acids (3); anions (2)	**11**
NIST	3254	Green tea (*Camellia sinesis*) leaves	Catechins (5); caffeine and theobromine; toxic elements (4); DNA sequence (identity)	Catechins (2), gallic acid, L-theanine, elements (5)	**21**
NIST	3255	Green tea (*Camellia sinesis*) extract	Catechins (7); caffeine and theobromine; toxic elements (2)	Catechins (2), gallic acid, L-theanine, theophylline, elements (5)	**21**
NIST	3256	Green tea-containing SODF	Catechins (6); caffeine and theobromine; gallic acid; toxic elements (4)	Catechins (1), L-theanine, theophylline	**16**
NIST	3234	Soy Flour	Elements (8); vitamins (2)	Elements (1); isoflavones (5); proximates (5) and fiber; amino acids (18)	**39**
NIST	3235	Soy Milk	Elements (8); vitamins (5)	Elements (1); vitamins (4); proximates (6), sugars (1); fatty acids (11); amino acids (16)	**52**
NIST	3236	Soy Protein Isolate	Isoflavones (6)		**6**
NIST	3237	Soy Protein Concentrate	Isoflavones (1)	Isoflavones (2)	**3**
NIST	3238	Soy-Containing SODF	Isoflavones (5)	Isoflavones (1)	**6**
NIST	3262	St John's Wort (*Hypericum perforatum*) Aerial Parts	Toxic elements (3); DNA sequence (identity)	Flavonoids/naphthodianthrones (5); chlorogenic acid; toxic elements (1)	**10**
NIST	3232	Kelp (*Thallus laminariae*) Powder	Elements (15) including toxic elements (4) and iodine	Elements (5), arsenic species (2), arsenosugars (3), vitamin K_1_ (3), proximates (5)	**33**
NRCC	GINX-1	North American Ginseng (*Panax quinquefolius*) Root Extract	Ginsenosides (7); elements (9)	Elements (9)^a^	**25**
NIST	3253	Yerba Mate (*Ilex paraguariensis*) Leaves	Polycyclic aromatic hydrocarbons (PAHs) (5)	PAHs (13); proximates	**23**
NIST	3299	Turmeric (*Curcuma longa*) Rhizome	Curcuminoids (3); toxic elements (3)	-	**6**
NIST	3300	Curcumin Extract of Turmeric (*Curcuma longa*) Rhizome	Curcuminoids (3)	-	**3**
NIST	3398	Ginger (*Zingiber officinale*) Rhizome	Toxic elements (3)	Gingerols (3) and shogaols (3); arsenic species (3)	**12**
NIST	3399	Ginger (*Zingiber officinale*) Rhizome Extract	Toxic elements (3)	Gingerols (3) and shogaols (3);	**9**
NIST	8650	Kudzu (*Pueraria Montana* var. *lobata*) Rhizome	-	Isoflavones (3); toxic elements (3); DNA sequence (identity)	**6**
NIST	3268	Kudzu (*Pueraria Montana* var. *lobata*) Rhizome Extract	Toxic elements (3); nutritional element (1)	Isoflavones (3)	**7**
NIST	3269	Kudzu-Containing SODF	-	Isoflavones (3)	**3**
NIST	3384	Asian Ginseng (*Panax ginseng*) Rhizome	Toxic elements (2)	Ginsenosides (7); toxic elements (1)	**10**
NIST	3385	Asian Ginseng (*Panax ginseng*) Rhizome Extract	Ginsenosides (6); DNA sequence (identity)	Ginsenosides (1); toxic elements (3)	**10**

a *Information values for four elements*.

**Table 2:** Currently available non-botanical dietary supplement matrix CRMs.

**Source**	**CRM No**.	**CRM description**	**Certified values**	**Reference values**	**Total values**
NIST	3280	Multivitamin/multielement tablets	Vitamins and carotenoids (13); elements (18)	Vitamins and carotenoids (4); elements (9)	**43**
NIST	3278	Tocopherols in edible oils	Tocopherols (4)		**4**
NIST	3275	Omega-3 and omega-6 fatty acids in fish oil	Fatty acids (31)	Fatty acids (23)	**54**
NIST	3275-1	Concentrate high in DHA	Fatty acids (9)	Fatty acids (7)	**16**
NIST	3275-2	Anchovy oil (high in DHA and EPA)	Fatty acids (11)	Fatty acids (7)	**18**
NIST	3275-3	Concentrate containing 60% long chain omega-3 fatty acids	Fatty acids (11)	Fatty acids (9)	**20**
NIST	3530	Iodized salt (Iodide)	Iodine (as iodide)	-	**1**
NRCC	VITA-1	Low-level multivitamin	Elements and element species (16); vitamins (1)	Elements (5)^a^; vitamins (7)^a^	**29**
NRCC	VITB-1	Elevated-level multivitamin	Elements and element species (16); vitamins (1)	Elements (5)^a^; vitamins (7)^a^	**29**
NIST	3279	Chromium-Containing Solid Oral Dosage Form	chromium	vanadium	**2**
NIST	8037	Krill Oil	-	fatty acids (22)	**22**

a *Informational values available for three elements and seven vitamins*.

**Table 3:** Selected in-development dietary supplement NIST materials.

**Candidate SRM description**	**Proposed constituents for value assignment**
Yohimbe (*Pausinystalia johimbe*) containing SODF	Yohimbine
Black Cohosh (*Actaea racemosa*) rhizome	Triterpene glycosides and toxic elements
Black Cohosh (*Actaea racemosa*) rhizome extract	Triterpene glycosides and toxic elements
Eluethero (*Eleutherococcus senticosus*) root	Eleutherosides and toxic elements
Eleuthero (*Eleutherococcus senticosus*) root extract	Eleutherosides and toxic elements
Ashwagandha (*Withania somnifera*) root	Withanosides and withanolides
Ashwagandha (*Withania somnifera*) root extract	Withanosides and withanolides
Kava (*Piper methysticum*)	Kava lactones

While the primary focus of the NIH-ODS/NIST collaboration has been to provide matrix SRMs for the dietary supplement community, several calibration solution SRMs were also developed by NIST between 2011 and 2016 for catechins, hypericin, organic acids, and isoflavones. Currently, the only botanical dietary supplement ingredient calibration solution available from NIST is SRM 3389 Ginsenosides Calibration Solution (47). Another calibration solution CRM for ginsenosides is also available from the National Research Council of Canada (the NMI for Canada) as well as three pure reference standards for constituents associated with plants used as ingredients in dietary supplements^13^ (see Table 4). In an effort to expand the availability of calibration CRMs for botanical dietary supplement ingredient markers, the AMRM Program engaged the private sector to produce these analytical resources, with CRMs for key constituents of kava (*Piper methysticum* G. Forst.) and ginger (*Zingiber officinale* Roscoe) recently available from Cerilliant/MilliporeSigma (48). Table 4 summarizes currently available, select calibration solution and pure chemical CRMs from NMIs and commercial sources, including those developed with support from NIH-ODS.

**Table 4:** Currently available pure material and calibration solution CRMs for botanical dietary supplement marker compounds.

**Source**	**CRM No**.	**CRM name**	**Certified values**
NIST	SRM 3389	Ginsenosides calibration solution^a^	Ginsenosides (6)
NRCC	MIGS-1	Multi-component ginsenoside calibration solution	Ginsenosides (7)
NRCC	BERB-1	Berberine chloride^b^	Berberine and berberine chloride purity^c^
NRCC	CANA-1	Canadine^d^	Canadine^c^
NRCC	HYDR-1	Hydrastine^b^	Hydrastine
Cerilliant	G-013	*Ginkgo biloba* terpene lactones mix	Ginkgolides (4) and bilobalide
Cerilliant	G-014	*Ginkgo biloba* flavonoids mix	Flavonoids (3)
Cerilliant	G-015	Ginseng ginsenosides mix	Ginsenosides (8)
Cerilliant	G-016	Green tea catechin mix	Catechins (7); caffeine
Cerilliant	G-027	Ginger gingerols and shogaols mix^a^	Gingerols (3); shogaols (3)
Cerilliant	K-007	Kava kavalactone mix^a^	Kavalactones (9)

a *Developed in collaboration with NIH-ODS*.

b *Berberine and hydrastine are naturally occurring isoquinoine alkaloids in several plant dietary supplement ingredients, including goldenseal*.

c *Information values for trace impurities*.

d *Canadine is a benzylisoquinoline alkaloid present in plants from the family Papaveraceae*.

## Analytical Principles for Chemical Composition Value Assignment

To provide true values for chemical content, CRM producers typically use orthogonal analytical methods, meaning the methods are based on different measurement principles, an approach that NP researchers can apply for greater confidence in their chemical characterizations. The assignment of certified values for the chemical composition of CRMs at NIST is based primarily on the agreement of results from multiple independent analytical methods. The development of the multiple independent methods concept for assigning certified values for trace elements in matrix SRMs has been described, along with a discussion of the importance of independence in the physical principle upon which the measurement is based and in sample preparation, standards, and calibration (49). For the determination of elements in matrix SRMs, the concept of using multiple independent methods is relatively straightforward because there are a variety of analytical techniques that are based on different measurement principles [e.g., inductively coupled plasma-optical emission spectroscopy (ICP-OES), ICP-MS, neutron activation analysis] and different sample preparation approaches are available (e.g., direct analysis of a solid sample or dissolve the matrix and analyze the resulting solution). For the determination of trace organic constituents, independence in the analytical method is achieved in the sample preparation (i.e., extraction, clean-up, and isolation of the compounds of interest) and the final chromatographic separation and detection (e.g., GC vs. LC and UV/fluorescence detection vs. MS or MS/MS detection) (28, 50). Independence is also incorporated in the quantification approaches used, including the use of isotopically labeled internal standards [i.e., isotope dilution (ID) approach] for both elements and organic constituents. If the results from the multiple independent methods agree, the possibility of undetected bias in the resulting certified value is minimized. Epstein summarized the historical development of the multiple independent methods approach for trace element determination (49), and Wise et al. have discussed the application of this approach for trace organic constituents (28, 50). In 2000 NIST formalized the approaches, or modes, for assigning values and established a hierarchy of values (denoted as certified, reference, and information) and associated confidence in their accuracy based on the various approaches used (51). This document was recently updated with numerous examples illustrating the implementation of various certification modes (52).

## Case Studies of Research Using Dietary Supplement Matrix CRMs

A number of research studies investigating the composition and health effects of dietary supplements have utilized RMs and CRMs to support measurement rigor and reproducibility. The case studies and figures described below describe selected examples of where the utilization of specific RMs facilitated innovative method development or empowered investigations by playing a key role in generating and interpreting novel research data.

### Method Development and Population Studies for Assessing DS Content and Exposure

NIST SRM 3280 Multivitamin/Multielement Tablet has been extensively used for method development applications and measurement verification of nutrient exposure assessments since its initial 2010 availability (Table 5). Van Berkel and coworkers used SRM 3280 in method development/validation studies for rapid and high-throughput determination of vitamins B_1_, B_2_, B_3_, B_5_, and B_6_ (56) and ascorbic acid and folic acid (59) using flow injection MS/MS without chromatographic separation and demonstrated good agreement with the certified values. Kakitani et al. validated an LC-MS/MS method for 15 water-soluble vitamins in dietary supplements and beverages by comparing results from the analysis of SRM 3280 (66). Chen et al. developed a single-laboratory validated method using LC with three different detection modes, i.e., diode array detection (DAD), fluorescence detection (FLD), and MS for the determination of seven B vitamins. SRM 3280 was used in the validation of the repeatability and ruggedness of the method; however, since this study preceded certification of the values for the vitamins, the method's accuracy was not assessed (53).

**Table 5:** Examples of reported use of SRM 3280 multivitamin/multielement tablets.

**References**	**Uses**			**Comments**
	**Method dev./valid**.	**QC**	**Novel Research**	
Roseland et al. (20)		X		Used as QC material to evaluate laboratory capabilities and measurement performance
Chen et al. (53)	X			Single-laboratory validation of HPLC-DAD method for water soluble vitamins in multivitamin tablets; SRM 3280 used for reproducibility assessment
Avula et al. (54)	X			Validation of ICP/MS method for 21 elements in dietary supplements; results for SRM 320 reported
Avula et al. (55)		X		Analyzed as control for determination of 16 elements in multivitamin supplements using ICP/MS
Bhandari and Van Berkel (56)	X			Validation of flow-injection MS/MS method for high-throughput determination of B vitamins in supplements
Matsumoto et al. (57)	X			Validation of LC-UV/visible method for vitamin B_12_ in MVM
Sullivan and Zywicki (58)	X			Results for the determination of iodine in SRM 3280
Bhandari et al. (59)	X			Validation of flow-injection MS/MS method for ascorbic and folic acid in multivitamin tablets; results compared with SRM 3280
Christopher and Thompson (60)	X			Determination of cadmium using ID-ICP/MS
Murphy and Vetter (61)	X			Determination of cadmium in dietary supplements
Raju et al. (62)	X			Method development for vitamin B_12_ using IC-ICP/MS
Yilmaz et al. (63)	X			Validation of solid phase extraction of Cu ions from high salt matrices prior to determination by flame atomic absorption spectrometry (FAAS); no results reported
Andrews et al. (64)		X		Investigated variability of vitamin D content in MVM products
Wolle et al. (65)	X			Extraction method development for determination of arsenic in dietary supplements using IC-ICP/MS
Kakitani et al. (66)	X			Validation of LC-MS/MS method for water soluble vitamins in dietary supplements and beverages; results reported for comparison
Pehrsson et al. (67)		X		Used for QC for determination of iodine content in food and dietary supplements
Qiu et al. (68), Novakova et al. (69)	X			Validation for flow-injection TiO_2_-mediated UV-photochemical volatile species generation atomic absorption spectroscopy (AAS) method for determination of selenium in supplements; comparison results reported
D'Ulivo et al. (70)	X			Validation of ID-LC-MS/MS method for determination of cyanocobalamin (vitamin B_12_)
Andrews et al. (14)		X		Used as QC material for analyses used to provide data for the Dietary Supplement Ingredient Database
White et al. (71)	X			Method development for cadmium in multivitamin supplements using ID-ICP/MS with coprecipitation schemes
Qiu et al. (68)	X			Single-laboratory validation study for vitamin B_12_ (cobalamin) using RPLC with DAD; results reported and compared
Begu et al. (72)	X			Validation of ICP/MS method for determination of arsenic and cadmium in salt matrix of multivitamin supplements using sequential coprecipitation
Crighton et al. (73)	X			Investigated the application of Direct Sample Analysis (DSA)-TOF for screening adulterated dietary supplements

*Uses include method development and/or validation and quality control (QC)*.

The development of non-microbiological assays to determine vitamin B_12_ (cyanocobalamin) in foods and DS has been the focus of several studies that utilized SRM 3280 to verify measurement accuracy. Chen et al. developed an LC-UV/Visible method to determine vitamin B_12_ in multivitamin/multielement DS (MVM) with improved efficiency through on-line sample clean-up, and they assessed accuracy, precision, recovery, limit of detection, and limit of quantification using SRM 3280 (74). Subsequent method development and validation by Matsumoto et al. analyzed SRM 3280 along with samples representative of multivitamin DS with or without elements and other NP ingredients like coenzyme Q10 to assess the accuracy and uncertainty range of their HPLC-UV detection method (57). D'Ulivo et al. developed a novel ID method for cyanocobalamin using an isotopically enriched ^13^C^15^N cyanocobalamin as the internal standard and validated the LC-MS/MS method using SRM 3280 (70). The validated ID-LC-MS/MS method was then employed to certify the content of cyanocobalamin in two multivitamin CRMs recently issued by the National Research Center of Canada (NRCC), i.e., VITA-1 and VITB-1. Qiu et al. utilized SRM 3280 in a single-laboratory validation study to demonstrate their HPLC-DAD method for determination of cobalamin in dietary ingredients and DS products (i.e., tablets, capsules, and chewable gels) achieved AOAC International Standard Method Performance Requirements (68). All of these method validation studies to determine vitamin B_12_ in SRM 3280 have provided valuable information toward assessing the true value, as shown in Table 6. The value for vitamin B_12_ in SRM 3280 was initially assigned using only results from two interlaboratory studies using microbiological assays and denoted as a reference value with a relatively large associated uncertainty (39%). However, this value was revised to 4.8 ± 1.0 mg/kg and upgraded to a certified value based on the combination of results from a NIST LC-ICP/MS method (4.51 ± 0.38 mg/kg). In the five method development studies in Table 6, the value for vitamin B_12_ ranged from 4.28 mg/kg to 6.02 mg/kg, with all methods reporting low Relative Standard Deviations (RSD). These results illustrate the analytical challenge associated with vitamin B_12_ measurements in MVM and emphasize the need to use a CRM to assess the accuracy of the analytical method.

**Table 6:** Values for vitamin B_12_ in SRM 3280 from different method development studies.

**References**	**Method**	**Value (mg/kg)**	***n* ^a^**	**Comments**
Chen et al. (74)	LC/UV	6.02 ± 0.05	15	Part of single laboratory validation study for method precision
Sander et al. (46)	Microbiological assays	4.9 ± 1.9	3,2	Value assignment based on two interlaboratory studies of 3 and 2 laboratories using microbiological assays
Wise and Phillips (75)	LC-ICP/MS	4.51 ± 0.38	10	Results used to update certified value
COA updated 2011		4.8 ± 1.0		Combined microbiological assay and LC-ICP/MS results for updated certified value
Matsumoto et al. (57)	LC-Visible	4.64 ± 0.11	24	
Raju et al. (62)	LC-ICP/MS	4.38 ± 0.05	2	
D'Ulivo et al. (70)	ID-LC-MS/MS	5.41 ± 0.18	4	Method used to certify two new MVM CRMs
Qiu et al. (68)	HPLC-DAD	4.28 ± 0.06	4	Single-laboratory validation study for vitamin B_12_ method

a *n = number of replicate measurements used to determine the value*.

The use of SRM 3280 for the determination of minerals and toxic elements (As, Cd) for both method development and validation has been reported by several researchers using ICP/MS (54, 55, 60, 71, 72) and AAS (63, 69). Wolle et al. used SRM 3280 for extraction method development in the determination of arsenic in dietary supplements (65). Sullivan and Zywicki used SRM 3280 in a single-laboratory validation study for an ICP/MS method for the determination of total iodine in various foods and DS (58). To assess precision and accuracy, they reported the individual measurements for 20 replicates ranging from 86 to 102% recovery resulting in a mean result of 125 ± 11 μg/kg (95% confidence interval) compared with the certified value of 132.7 ± 6.6 μg/kg; ruggedness was assessed by comparing results from a second analyst for six replicates [mean of 137 μg/kg (RSD = 3.8%)], and combined results were 128 μg/kg (RSD = 5.9%, *n* = 26).

The consumption of DS contributes significantly to vitamin and mineral intake in U.S. populations, and clinical studies of dietary interventions should take this baseline exposure into account when designing trials and interpreting results. The Dietary Supplement Ingredient Database (DSID), a collaborative effort between NIH-ODS and U.S. Department of Agriculture (USDA), supports research on DS health effects by providing analytically determined estimates for the nutrient and phytochemical content of representative DS products marketed to certain populations, such as MVMs for adults or children or prenatal supplement products. DSID investigations routinely use NIST SRMs to verify laboratory measurements of vitamins, minerals, fatty acids, toxic elements, and botanical phytochemicals. DSID studies have found wide ranges of ingredient content variability compared to DS product label claims and trends of concentration overages (14, 64, 76), and have investigated if certain constituents, such as caffeine, may reach levels of concern (77). By using CRMs, DSID researchers thus established measurement confidence in their conclusions on whether exposures from DS may approach Tolerable Upper Intake Levels for nutrients or otherwise cause safety concerns for NP constituents.

### Vitamin D and 25-Hydroxyvitamin D_3_

A USDA-coordinated study to assess the measurement capabilities for vitamin D_3_ and its primary metabolite, 25-hydroxyvitamin D_3_ [25(OH)D_3_], in food and dietary supplement matrices (78) provides an excellent example of using existing matrix RMs to assess analytical methods for analytes that do not already have value assignments. Vitamin D_3_ and vitamin D_2_ are metabolized in animals to 25(OH)D_3_ and 25(OH)D_2_, respectively, which may be present in animal tissues along with unmetabolized vitamin D_3_ and vitamin D_2_. Studies suggest that 25(OH)D_3_ may be more potent than vitamin D in elevating serum levels of total 25(OH)D, which is the sum of 25(OH)D_3_ and 25(OH)D_2_ and the common clinical marker for vitamin D status (78). Therefore, accurate measurement of 25(OH)D in food and dietary supplements is critical to provide reliable estimates of vitamin D intakes. The goal of the USDA study was to assess the capabilities of selected laboratories to measure 25(OH)D in food and dietary supplement matrices and the potential use of these materials as reference materials [i.e., assign values for vitamin D and 25(OH)D]. Although no DS matrix RMs were included in the study, three existing food matrix SRMs that did not have values assigned for vitamin D or 25(OH)D were included, namely bovine liver (SRM 1577c), whole egg powder (SRM 1845a), and meat homogenate (SRM 1546a). Results from this study were later used by NIST, in conjunction with results from an ID-LC-MS/MS method at NIST, to assign values for vitamin D_3_ and 25(OH)D_3_ in these food matrix SRMs, the first CRMs with values assigned for 25(OH)D_3_ (79).

### Green Tea SRMs in Metabolomic Studies

Excellent examples of the use of CRMs in botanical research studies are found in recent studies leveraging metabolomics in characterizing *Camellia sinensis* (L.) Kuntze preparations and assessing variability among products. Punyasiri et al. (80) used NIST SRM 3254 (*C. sinensis*) Green Tea Leaves to aid their development of optimized sample preparation approaches for metabolic profiling that mitigated degradation of key flavonoids; the matrix SRM's certified values for major catechins enabled confirmation of their measurement accuracy and precision. Kellogg et al. (81) used SRM 3254 in a study comparing conventional solvent maceration vs. accelerated solvent extraction for metabolomic characterization; however, rather than comparing their measurements to certified values, they instead used the SRM as one of four samples to evaluate differential catechin extraction by the two different methods. In a study by Tian et al., SRM 3254 was included as one of five green tea samples to demonstrate the feasibility of a biochemometric approach that combined metabolic profiling with a bio-fractionation assay to identify intestinal UDP-glucuronosyltransferase inhibitors in green tea, and the results for the SRM were found to be qualitatively similar to the other samples (82).

**Figure 2:**
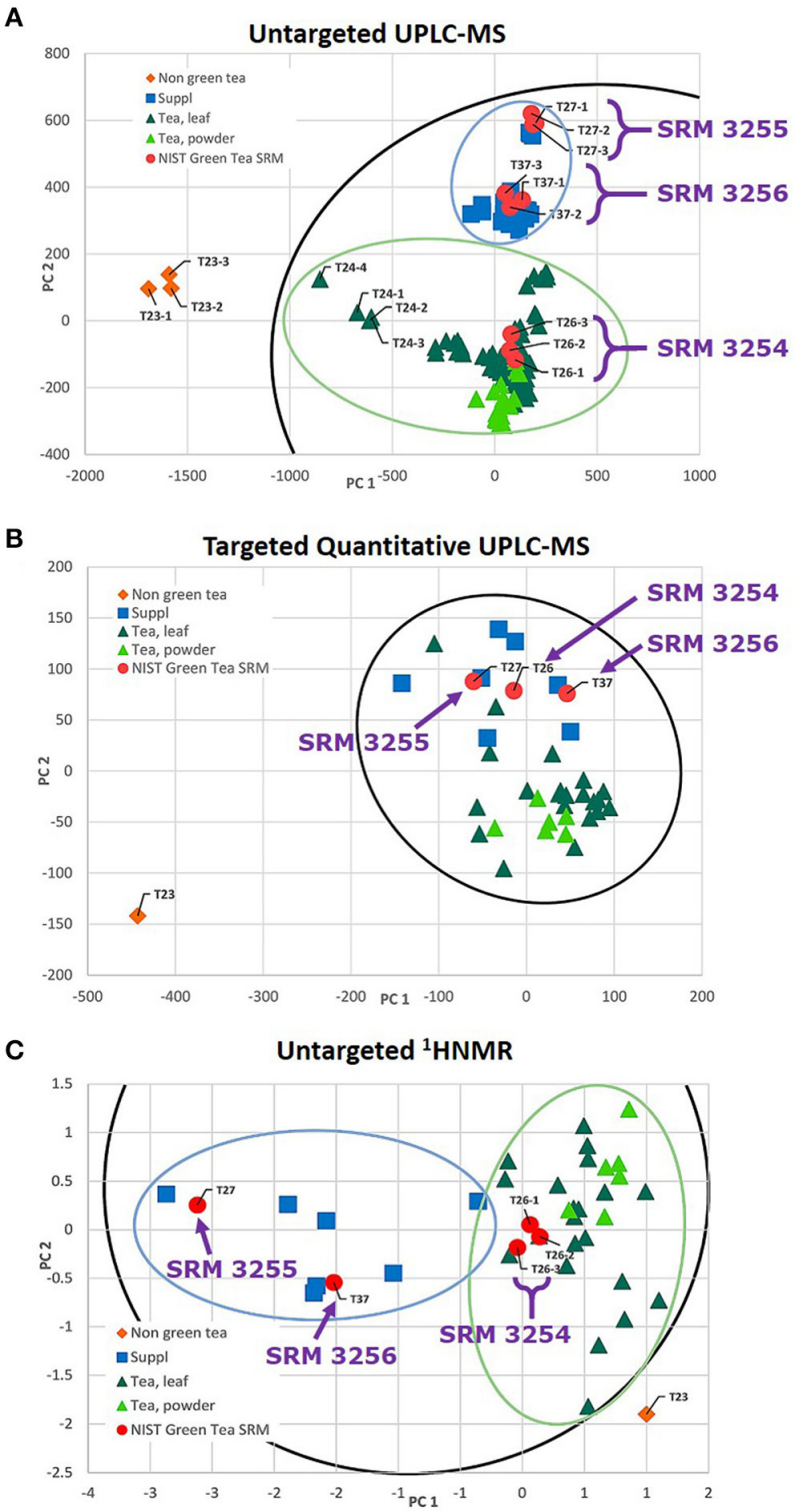
Principal component analysis (PCA) score plots of green tea samples drawn with Hotelling's 95% confidence ellipse for **(A)** untargeted UPLC-MS, **(B)** targeted quantitative UPLC-MS, and **(C)** Untargeted ^1^HNMR. Data points representing triplicate green tea samples were closely clustered, and distinct clusters were observed between green tea supplements (denoted as Suppl), green teas (leaf and powder), and the negative control (turmeric-ginger T23 denoted as non-green tea). Representative samples are highlighted, including NIST SRM 3254 (T26), SRM 3255 (T27), and SRM 3256 (T37), to demonstrate the reproducibility of the extraction and analytical protocol. Adapted with permission from Kellogg et al. (83); further permissions related to this excerpted material should be directed to the American Chemical Society.

In a study that compared metabolomic approaches to evaluate phytochemical variability, Kellogg et al. used three NIST green tea SRMs (leaves, extract, and SODF) as “positive controls” as part of their evaluation of untargeted UPLC-MS, targeted quantitative UPLC, and untargeted ^1^H nuclear magnetic resonance spectroscopy (NMR) to assess chemical similarity and variability among 34 different commercial green tea products (83). Additionally, SRM 3254 was used to demonstrate catechin extraction reproducibility and evaluate hot water vs. methanol extraction efficiency. All three green tea SRMs were analyzed as part of the sample set to determine a set of 16 targeted marker compounds. Principal component analyses (PCA) of the three metabolic approaches found that the various green tea samples clustered with the respective matrix-matched SRMs (Figure 2) and demonstrated that the untargeted MS-based metabolomics was more effective in discriminating among the classes of green tea products than either untargeted UPLC-MS or ^1^HNMR metabolomics. Another key finding was that had they relied on PCA analysis based solely using targeted marker compounds, which represent only a subset of the chemical diversity of the various green tea samples, they might have overlooked the actual chemical dissimilarity of the various products. Leveraging the full NIST suite of green tea SRMs, which represented the three categories of products (ground leaf, extract, and SODF), was instrumental in confirming this conclusion.

**Figure 3:**
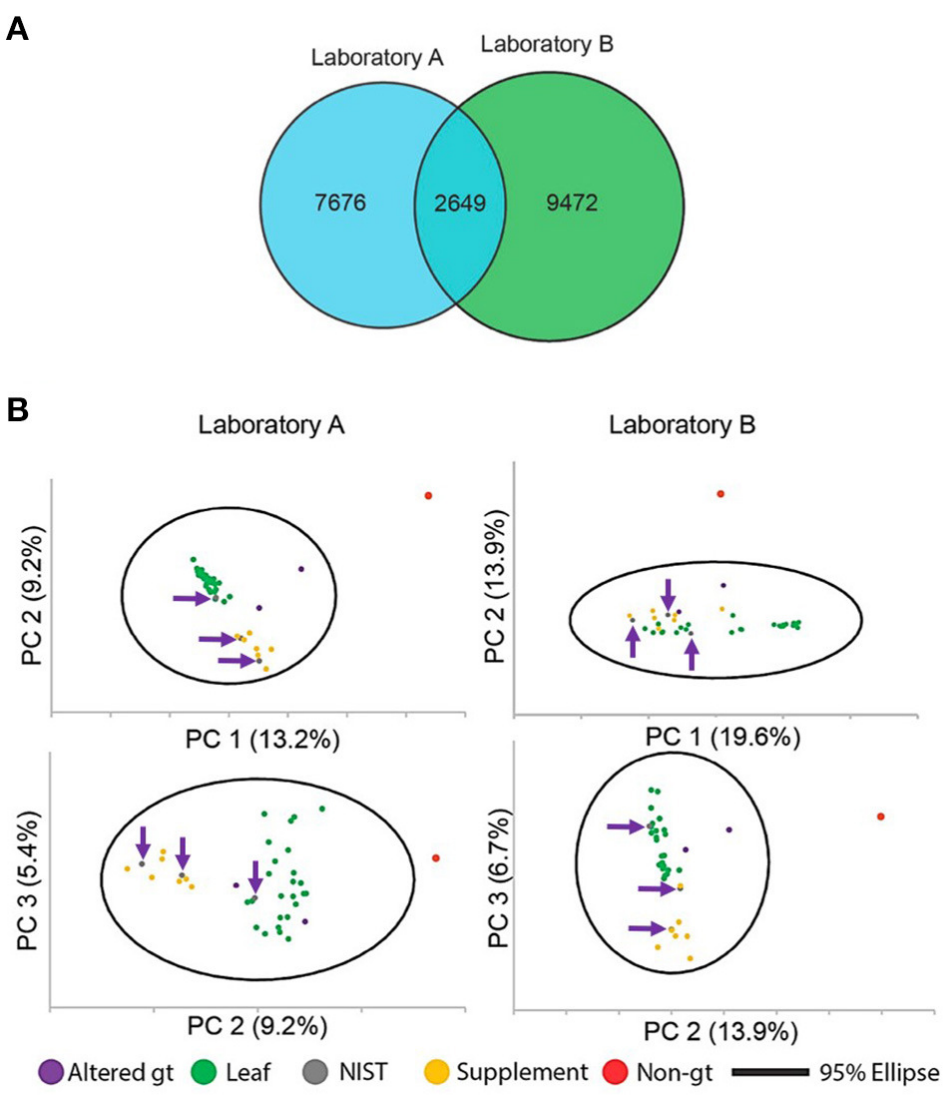
Comparison of MS features derived from untargeted MS analyses by two independent labs for green tea [*C. sinsensis* (L.) Kuntze] samples and unknowns, including 27 leaf or powder ingredients, seven supplement products, a non-green tea turmeric-ginger tea (non-gt), and three NIST green tea SRMs (SRM 3254 leaf, SRM 3255 extract, and SRM 3256 SODF). **(A)** Venn diagram of unique feature list counts and shared features between laboratories A (blue) and B (green). **(B)** Principal component analysis (PCA) score plots drawn with Hotelling's 95% confidence ellipse from Laboratory A (left) and Laboratory B (right). Top plots are PC1 vs. PC2; bottom plots are PC2 vs. PC3. Green tea ingredients (leaves and powders) were qualitatively separated from DS products, and NIST SRMs (gray dots, highlighted with purple arrows) were plotted within matrix matched clusters. Reprinted (adapted) with permission from Clark et al. (84). American Chemical Society.

In a follow-up study from Cech and colleagues, Clark et al. employed the same green tea commercial samples and SRMs to conduct an interlaboratory comparison of untargeted MS data sets to investigate underlying causes for measurement variability (84). To determine if there was experimental value in performing replicate extractions and replicate injections of the same botanical extract, the authors extracted metabolites from SRM 3254, and the samples were analyzed in two different laboratories using the same LC column, LC gradient, and mass spectrometer acquisition parameters but on different mass spectrometer platforms. The study showed that a significant portion of the features detectable in single replicate measurements are not observed in two out of the three replicates, suggesting that there is little value to conducting replicate injections of replicate extracts if features that appear only once are omitted from analyses. Clark et al. hypothesized that variations in the generated composition feature lists between the two instruments were driven by differences in feature formation for the same molecule sets. To address this, they expanded their study to the larger sample set of 37 green tea samples, including the three NIST green tea SRMs. The results are summarized in Figure 3 with a Venn diagram and PCA score plots. As in the previous study, the PCA score plots were successful in discriminating among the three sample types with each of the three SRMs in the expected matrix category. The study of Clark et al. emphasizes that “untargeted metabolomics feature lists are not a description of the chemical composition of the sample, but rather an instrument-specific snapshot of how the chemical entities in the sample respond to the analysis by the particular mass spectrometer” (84). This conclusion supports the need for metabolomic researchers to use commonly available and well-characterized samples, such as the NIST SRMs, to facilitate the comparison of results among laboratories.

### Fish and Botanical Oil SRMs for Determination of Fatty Acids

**Figure 4:**
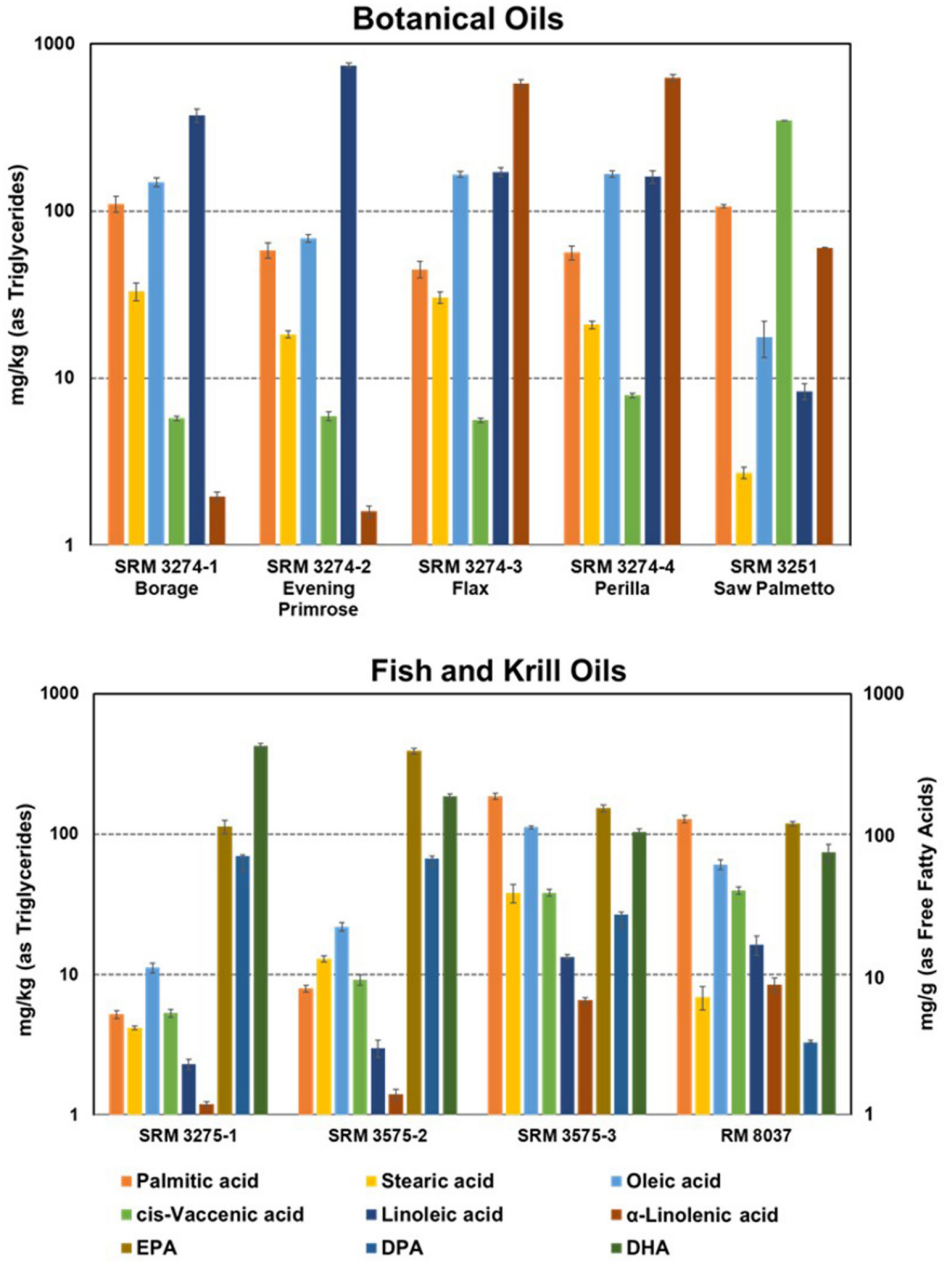
Bar graphs of the distribution of mass fractions of selected fatty acids (as triglycerides) in marine and botanical oil SRMs and RMs. Note the logarithmic scale for the mass fractions, and for NIST RM 8037 Krill Oil the units are mg/g (as free fatty acids). Error bars are the expanded uncertainties of the certified and reference values with 95% confidence. Adapted and reprinted with permission from Springer, *Anal. Bioanal.Chem*., Wise and Phillips (75).

The determination of fatty acids using GC with flame ionization detection (FID) and GC-MS are relatively mature measurement techniques in food and dietary supplement analysis, and as a result, matrix-based CRMs to support these analyses have been available since the late 1990's (75). With the importance of fatty acids in nutrition studies, particularly omega-3 and omega-6 compounds, SRM 3274 Botanical Oils Containing Omega-3 and Omega-6 Fatty Acids and SRM 3275 Omega-3 and Omega-6 Fatty Acids in Fish Oil were developed as suites of four and three different mixtures of botanical and fish oils, respectively, to provide different levels and different ratios of the individual fatty acids. A krill oil material, which represents a different matrix used as dietary supplements and has mass fractions of fatty acids a factor of 1,000 greater than the fish oil materials, was issued in 2020 as RM 8037. The relative mass fractions of the botanical and marine oil SRMs and RM are shown in Figure 4 (85).

SRM 3275 has found wide use to validate methods and serve as a QC material as demonstrated by Srigley and coworkers (86–89) at the FDA reporting several studies using SRM 3275 for validation of methods. In a study to evaluate the overall fatty acid composition of 46 marine oil omega-3 supplements, Srigley and Rader analyzed SRM 3275 (all three levels) using GC-FID on a novel ionic liquid phase column (90). In addition to comparisons of their results for 21 fatty acids to the assigned values in SRM 3275, with excellent agreement, the authors also showed GC-FID chromatograms for SRM 3275 on this novel GC stationary phase, thus providing valuable information for comparison with separations on other stationary phases and with other researchers. Li and Srigley (87) later validated a novel method for the quantification of long-chain omega-3 polyunsaturated fatty acids in gummy dietary supplements using SRM 3275 and compared their results for eicosapentaenoic acid (EPA) and docosahexaenoic acid (DHA) with the certified SRM values with agreement in all three levels within 5%. Karunathilaka et al. used the fish oil SRM to validate a method for rapid classification and quantification of marine oil omega-3 supplements using Attenuated total reflection—Fourier transform infrared spectroscopy (ATR-FTIR), Fourier transform—near infrared spectroscopy (FT-NIR), and chemometrics (86, 88). In an evaluation of four ionic liquid columns for rapid analysis or the improved resolution of long-chain methyl and ethyl esters of omega-3, omega-6, and additional positional isomeric and stereoisomeric blends, Weatherly et al. used SRM 3275 for method validation (91). Khoomrung et al. compared results from a new fast preparation of fatty acid methyl esters by microwave-assisted derivatization with the certified values from SRM 3275-2 for method validation (92).

Ahn et al. from the Korea Research Institute of Standards and Science used SRM 3274 to validate their ID-GC-MS candidate reference method for the determination of three essential fatty acids (linoleic, α- and γ-linolenic acid) in supplement oil products (93). The results for the three fatty acids in the borage, evening primrose, and flax oil were in excellent agreement with the SRM certified values. Although not intended specifically for fatty acid determinations, two saw palmetto [*Serenoa repens* (W. Bartman) Small] matrix SRMs have been available for over a decade (SRM 3250 Saw Palmetto Fruit and SRM 3251 Saw Palmetto Extract) with values assigned for phytosterols and fatty acids (94, 95). As an oil extract, SRM 3251 has been used for QC in the determination of both phytosterols and fatty acids in saw palmetto supplements (96) and in virgin olive oil (97).

### Authentication of Black Cohosh

The value of matrix RMs in the development of novel methods for botanical characterization and identification is demonstrated in studies using chemical fingerprinting approaches for the authentication of black cohosh (*Actaea racemosa* L.). Investigations led by the USDA Methods and Application of Food Composition Laboratory utilized authenticated reference materials from multiple sources, including NIST candidate SRMs, for *A. racemosa* and four related species (*A. dahurica, A. pachypoda, A. podocarpa*, and *A. foetida*) in the development of methodological approaches for authentication that used chemical analyses such as LC, MS, or NMR combined with PCA and soft independent modeling of class analogy (SIMCA) (98–100). These data demonstrated an effective approach to differentiate *A. racemosa* roots and rhizomes from those of other related species and possible contaminants using statistical models built from the characterization of the authenticated RMs (98). Detailed assessments of the chemical fingerprints lead to the identification of ester and amide derivatives of hydroxycinnamic acids as novel marker compounds for authentication (99, 100). Therefore, using authenticated plant material RMs in these innovative statistical models supported rigorous non-targeted examinations of commercially available *Actaea* ingredients. These models for *Actaea* species discrimination by hydroxycinnamic acids provided good sensitivity and accuracy for plant materials. Notably, using these models for authenticity predictions for finished supplement products required targeted profiling of stable marker compounds, since processing into final dosage forms introduces additional chemical variation. Furthermore, while the related *Actaea* species could be differentiated from one another, analyses of the different RMs for *A. racemosa* rhizomes did not result in a single cluster in the PCA modeling. Instead, several factors such as growing location, harvest conditions, handling, or post-harvesting processing or storage conditions were suggested to result in sufficient chemical variation even among authenticated RMs. These studies serve to illustrate important points. First, where possible, employing orthoganol methods is a powerful approach for botanical authentication. Second, phytochemical variation is to be expected in botanical preparations and must be accounted for by any model that attempts to establish authenticity. Third, an authenticated RM or standard that is broadly available to the research community can help validate techniques that identify the genus and species or plant part. Researchers should be mindful, however, that any given single RM does not represent all possible inherent biological, environmental, and processing variability.

### Assessing Botanical Variability, Product Composition, and Formulation Performance

In addition to the identification and authentication of NPs, matrix-matched RMs are also valuable in validating quality control measurements and the detection of adulteration or contamination, essential considerations when designing intervention studies of dietary supplement health effects. Chromatographic profiling of phytochemicals, whether LC or high-performance thin-layer chromatography (HPTLC), is a well-established approach to querying the quality of botanical preparations, and RMs are routinely employed as comparators to experimental samples with an unknown provenance and/or quality. For example, pharmacopeial standards and CRMs were used as references in an investigation of *Ginkgo biloba* L. leaf and extract products that found the majority of samples contained elevated levels of quercetin and/or rutin, or low levels of marker metabolites when compared with chemically well-characterized reference standards (101). Importantly, the ability to match *G. biloba* leaf products to a leaf CRM and the extract products to an extract CRM fostered a higher level of confidence in the phytochemical profile comparisons.

Recently there has been an increased push for the application of more non-targeted and orthogonal methods in natural product quality control since assessments focusing on a limited number of chemical constituents or based on insufficiently distinct profiles can be prone to false conclusions. Here again, matrix-based CRMs offer valuable benefits to researchers. Beyond their role in confirming the presence and appropriate amounts of known compounds, RMs can support the systematic establishment of distinct chromatographic profiles for specific botanical preparations. For example, Napolitano et al. utilized NIST SRM 3255 (Green Tea Extract) to evaluate a novel quantitative ^1^H NMR (qHNMR) method and an orthogonal traditional LC-MS/MS method for multi-targeted determination of major catechins (102). Comparison *via* the SRM demonstrated agreement of the two methods for catechin measurements, and overall the study highlighted potential benefits of incorporating qHNMR analyses into natural product authentication and characterization. In another example, Harnly and colleagues used pharmacopeial standards and CRMs in their determination of chromatographic profiles of *G. biloba* leaves and processed materials that included over 40 flavonoids and terpene lactones, more than 20 of which were newly identified as *G. biloba* constituents (103). In a subsequent study, CRMs were included among authenticated samples to create a one-class SIMCA modeling approach that could easily detect adulteration with isolated phytochemicals like rutin and quercetin (104). Furthermore, these studies illustrated how the content of certain *G. biloba* phytochemicals, specifically biflavones, is differentially affected by processing during extraction and product manufacture and how excipients in formulations can prohibit the detection of adulterants by some analytical methods.

DSID studies used the suite of NIST green tea SRMs as analytical quality control materials to assure rigor and reproducibility in measurements of 32 different commercial products that determined percent differences between labeled and actual catechin content (105). DSID studies also leveraged the certified values for catechins in NIST green tea SRMs as measurement controls in experiments that quantified the extent to which different dosage forms disintegrated and how they affected catechin dissolution (106). These DSID studies highlight the importance of assessing formulation performance and considering the bioavailablity of the constituents when designing clinical intervention studies of dietary supplements.

### Dietary Supplement Laboratory Quality Assurance Program

In conjunction with the NIH-ODS, NIST established a Dietary Supplement Laboratory Quality Assurance Program (DSQAP)^14^ in 2007 to assist laboratories in improving measurements of active and marker compounds, nutritional elements, toxic elements, organic nutrients (e.g., vitamins and carotenoids), and contaminants in DS and food matrices. In the DSQAP, participating laboratories analyze unknown DS and food samples provided by NIST, and the results are then compared with either NIST assigned target values or a laboratory consensus value from the study (107, 108). NIST conducted 15 exercises from 2007 through 2017, typically consisting of five or six studies each, and thus covered over 90 different analyte/matrix combinations. In 2017, DSQAP was incorporated into a restructured Health Assessment Measurements Quality Assurance Program (HAMQAP)^15^, which expanded focus to include analysis of samples representative of both human intake (i.e., food and dietary supplements) and output (i.e., blood, serum, urine). From 2017 through 2021, HAMQAP conducted six exercises which have included ~34 studies that have increased emphasis on analyte/DS matrix combinations relevant to emerging research on DS metabolism and health effects. By participating in the DSQAP and HAMQAP, laboratories can demonstrate and assess their measurement capabilities and accuracy by comparison with NIST assigned or study consensus results.

**Figure 5:**
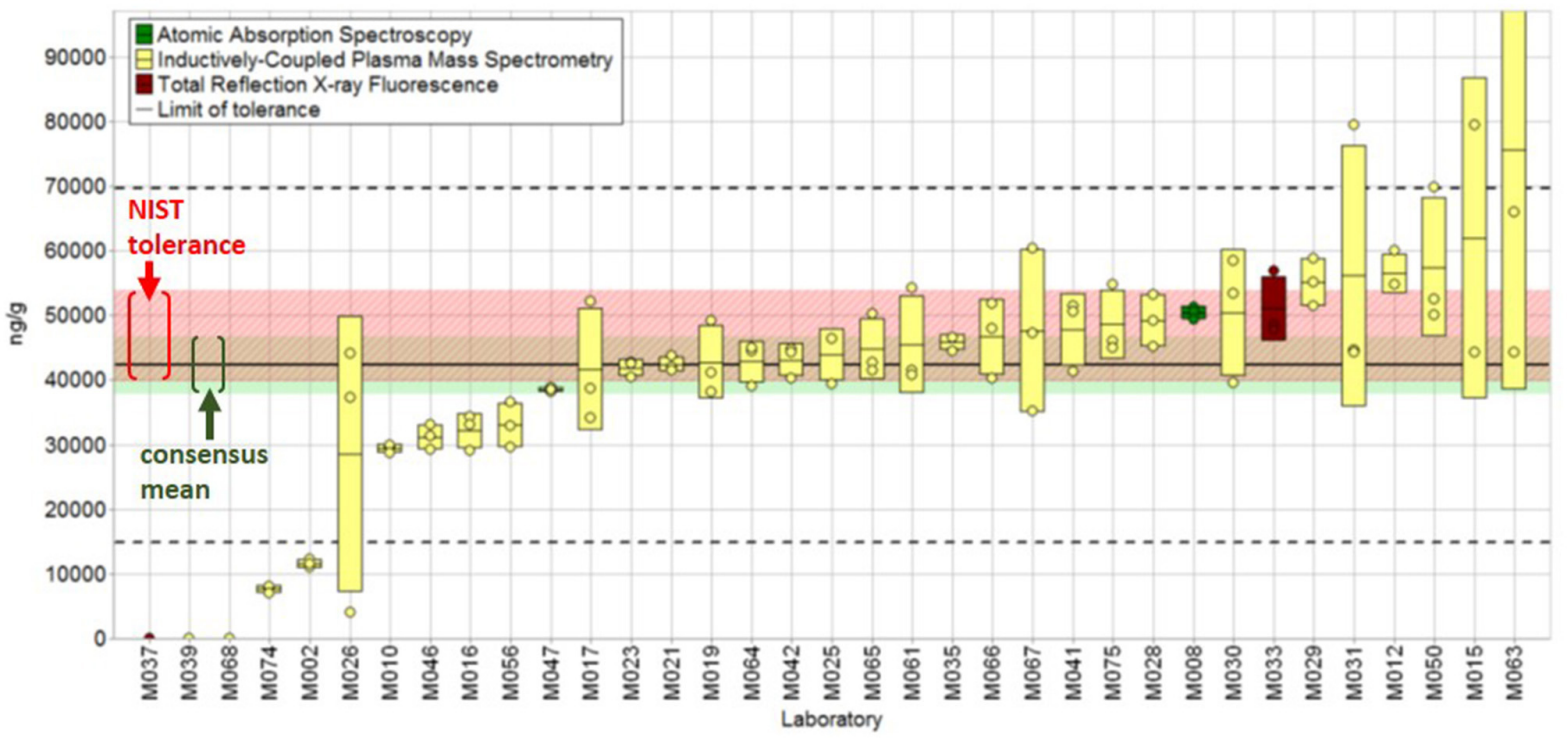
Total arsenic (ng/g) in NIST SRM 3398 Ginger (*Z. officinale* Roscoe) Rhizome. Individual laboratory data are plotted (circles) with standard deviation (*n* = 3, rectangle) in order of increasing magnitude. The color of the data rectangle indicates the analytical method employed. The solid black line is the consensus mean, and the green shaded region represents the consensus mean bounded by twice the consensus standard error. The black dashed lines represent the consensus range of tolerance calculated as the values above and below the consensus mean that result in an acceptable *Z'*_*comm*_ score. The red shaded region represents the NIST range of tolerance, which encompasses the NIST-determined value bounded by twice its uncertainty and represents the range that results in an acceptable *Z'*_*NIST*_ score. For a detailed discussion of the statistical treatment of the results, see Phillips et al. (109). Adapted with permission from Phillips et al., NISTIR 8203, 2018.

In the majority of the QAP studies, NIST SRMs or candidate SRMs (i.e., an SRM currently in progress that may or may not have values assigned) are distributed for analysis as unknown or known samples. When a candidate SRM is used, the results from the interlaboratory study may be used in combination with NIST measurements to assign the certified values. Several DS matrix SRMs have been used numerous times in these studies, including the multivitamin/multielement tablets, green tea, and fish and botanical oil materials (e.g., see NISTIR^16^ 7997, 8203, and 8308). Studies for the determination of toxic elements (arsenic, cadmium, lead, and mercury) in DS, particularly botanical matrices, have high levels of participation with 20–30 laboratories typically submitting results, in part because such analyses are routinely required to verify the safety of raw materials intended for use in the manufacture of dietary supplements. Representative results from a DSQAP study for the determination of arsenic in ginger rhizome (SRM 3398) are illustrated in Figure 5 with laboratory results presented from lowest to highest with color-coding to denote the different analytical techniques used (the majority use ICP/MS) with the consensus value with tolerance limits indicated.

**Figure 6:**
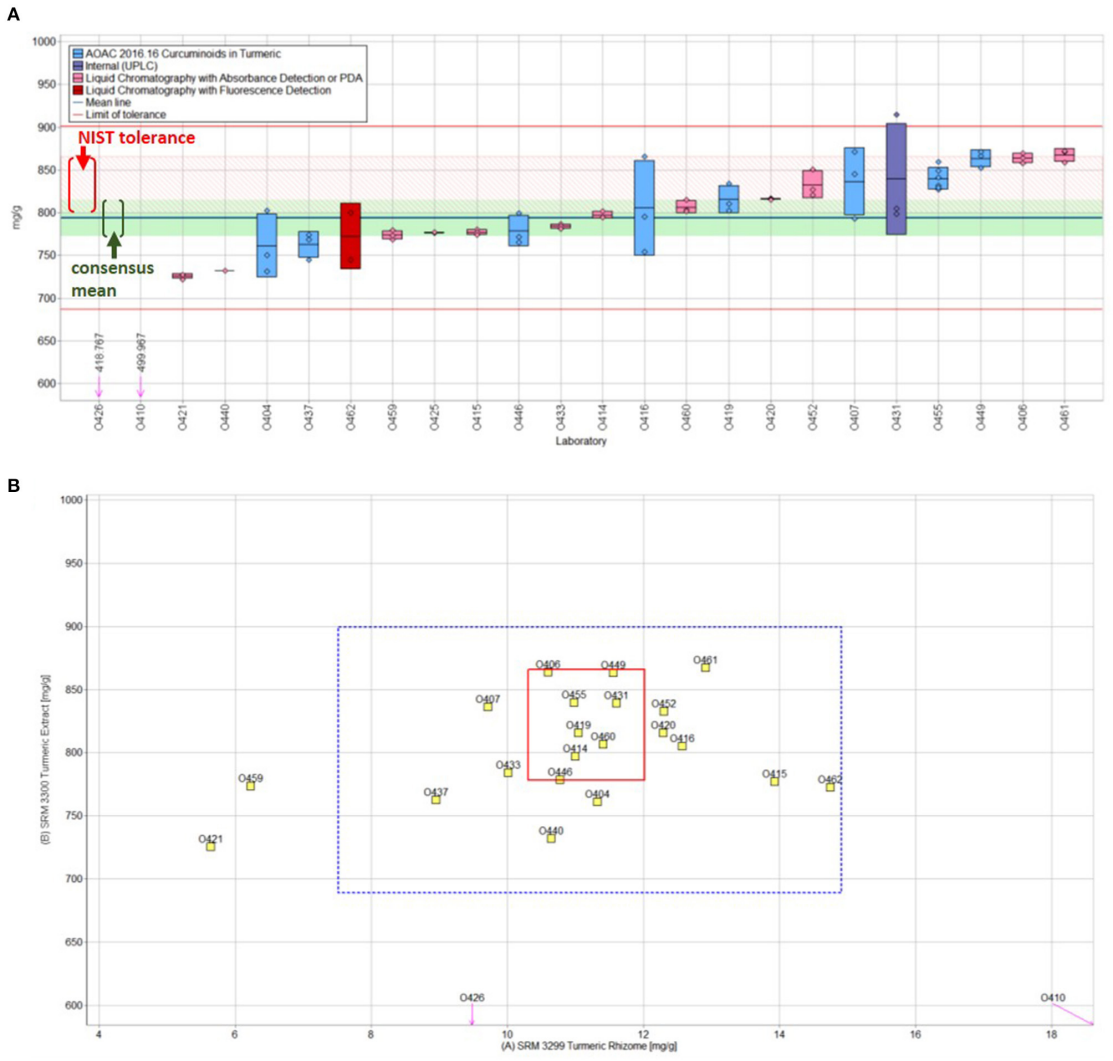
**(A)** Curcumin (ng/g) in NIST SRM 3300 Turmeric (*C. longa* L.) Rhizome Extract. Individual laboratory data are plotted (circles) with standard deviation (*n* = 3, rectangle) in order of increasing magnitude. The color of the data rectangle indicates the analytical method employed. The solid blue line is the consensus mean, and the green shaded region represents the 95% confidence interval for the consensus mean. The solid red lines represent the consensus range of tolerance calculated as the values above and below the consensus mean that result in an acceptable *Z'*_*comm*_ score. The red shaded region represents the NIST range of tolerance, which encompasses the NIST-determined value bounded by twice its uncertainty and represents the range that results in an acceptable *Z'*_*NIST*_ score. **(B)** Laboratory means for curcumin in NIST SRM 3299 Turmeric Rhizome and SRM 3300. The laboratory mean for the turmeric rhizome is compared to the mean of the extract for each laboratory. The solid red box represents the NIST range of tolerance for the two turmeric SRMs, rhizome (x-axis) and extract (y-axis), as the dotted blue box represents the consensus range of tolerance described for **(A)**. For a detailed discussion of the statistical treatment of the results, see Barber et al. (110). Adapted with permission from Barber et al., NISTIR 8266, 2019.

A similar plot is shown in Figure 6 for the determination of curcumin in turmeric extract (SRM 3300) and demonstrates excellent agreement of the study consensus value (820 ± 22 mg/g) and the NIST target value (822 ± 22 mg/g). The determination of curcuminoids in turmeric samples was the focus of two studies in DSQAP Exercise M (109) and Exercise O (110), with SRMs 3299 and SRM 3300 analyzed in both exercises. The second study for curcuminoids in turmeric was conducted with the intent of providing reproducibility and accuracy data to support moving the method for quantification of curcuminoids to final action status for AOAC *Official Method of Analysis (OMA)*. A total of eight participants used the AOAC OMA 2016.16 HPLC-DAD method for the quantification of curcuminoids (blue data rectangles in Figure 6), and these DSQAP results were reported by Mudge et al. as part of a multi-lab method validation study (111). The results for the two curcuminoid exercises are summarized in Table 7 and demonstrate the significant improvement in the overall performance of the laboratories as indicated by standard deviations of the consensus values decreasing from 17–20% to 3.6–5.7% for the turmeric rhizome and from 11–34% to 1.9–3.4% for the turmeric extract. A similar improvement is also observed for the results from two studies of the measurement of catechins in the green tea extract (SRM 3254) (see Table 7), with improvements from 10 to 72% in the 2012 study (DSQAP Exercise I) to 1–11% in the 2020 study (HAMQAP Exercise 5). In particular, results for the participant labs' measurement of epigallocatechin gallate (EGCG) improved more than 10-fold, as indicated by the reduction in the standard deviation of the consensus means between the two studies.

**Table 7:** Results for determinations of curcuminoids in turmeric and catechins in green tea in multiple QAP exercises.

**Curcuminoids**	**Target**		**DSQAP exercise M (*****n*** **=** **17–23)**		**DSQAP exercise O (*****n*** **=** **22–25)**	
	**SRM 3299**	**SRM 3300**	**SRM 3299**	**SRM 3300**	**SRM 3299**	**SRM 3300**
Bisdesmethoxycurcumin	3.390 ± 0.054	18.25 ± 0.49	3.23 ± 0.66 (20)	16.2 ± 5.5 (34)	3.16 ± 0.16 (5.7)	17.3 ± 0.58 (3.4)
Desmethoxycurcumin	3.634 ± 0.64	117.1 ± 1.2	3.26 ± 0.54 (17)	116 ± 13 (11)	3.63 ± 0.13 (3.6)	117 ± 2.2 (1.9)
Curcumin	11.17 ± 0.21	822 ± 11	11.6 ± 2.1 (18)	801 ± 123 (15)	11.20 ± 0.43 (3.8)	822 ± 22 (2.7)
**Catechins**	**Target**		**DSQAP exercise I (*****n*** **=** **17–28)**		**HAMQAP exercise 5 (*****n*** **=** **6–12)**	
	**SRM 3255**					
Catechin	8.88 ± 0.90		9.84 ± 2.37 (24)		7.95 ± 0.88 (11)	
Epicatechin	45.8 ± 6.5		43.2 ± 5.8 (13)		38.3 ± 2.1 (5.5)	
Epicatechin gallate	97.2 ± 7.6		95.1 ± 13.4 (14)		94.9 ± 3.5 (3.7)	
Epigallocatechin	79.2 ± 6.3		62.9 ± 34.8 (55)		82.4 ± 6.6 (8.0)	
Epigallocatechin gallate	409 ± 18		408 ± 39 (10)		406 ± 2.5 (0.6)	
Gallocatechin	21.3 ± 1.6		28.0 ± 20.2 (72)		19.8 ± 1.9 (9.6)	
Gallocatechin gallate	37.8 ± 1.9		43.1 ± 10.6 (25)		42.8 ± 1.5 (3.5)	

*Target (NIST-assigned) values and participant consensus (QAP exercise) values are listed for distributed SRMs. The range for the number of labs which reported data for individual analytes is noted (n); the uncertainty for each result is the standard deviation; the percent RSDs of the consensus values for each analyte are indicated in parentheses*.

The QAP exercises provide valuable information regarding the current state of measurement capabilities of laboratories and the need for CRMs for specific analyte/matrix combinations. In addition, laboratories participating in the QAP are introduced to the availability of dietary supplement matrix SRMs and the benefit of using them as part of their laboratory quality control procedures.

### Botanical Product Safety Assessments

Perhaps the most direct application of natural product reference materials to public health research is their use in safety evaluations. Whether an experimental natural product preparation will be used in *in vitro* cellular models or administered to animals or human subjects, it is incumbent upon researchers to assess the test article for known contaminants. First and foremost, when used as controls, reference materials enable researchers to confirm that their methods are fit for purpose for the detection of known toxins such as toxic metals, pesticides, or certain microbes and phytochemicals or secondary metabolites. Second, reference materials have been used as comparators for chemical composition in investigations of natural product preparations with putative health risks or unknown hazard profiles.

Analyses for known toxic constituents, whether from natural plant sources (e.g., pyrrolizidine alkaloids) or those incurred from the environment or industrial processing (e.g., mercury or lead), often must be performed quantitatively to assure safety through compliance with regulatory limits. In this context, the use of an appropriate CRM offers a clear advantage over less characterized qualitative standards. Investigations for the presence of toxic elements in NPs are routinely published in the peer-review scientific literature, and the utilization of CRMs with value assignment(s) for the toxin(s) of interest supports increased experimental rigor. A survey on the presence or absence of hazardous constituents in commercial dietary supplement products or traditional medicine preparations, and its subsequent implications on safety, has a higher level of confidence when CRMs are used to confirm measurement accuracy and reliability (112–114).

**Figure 7:**
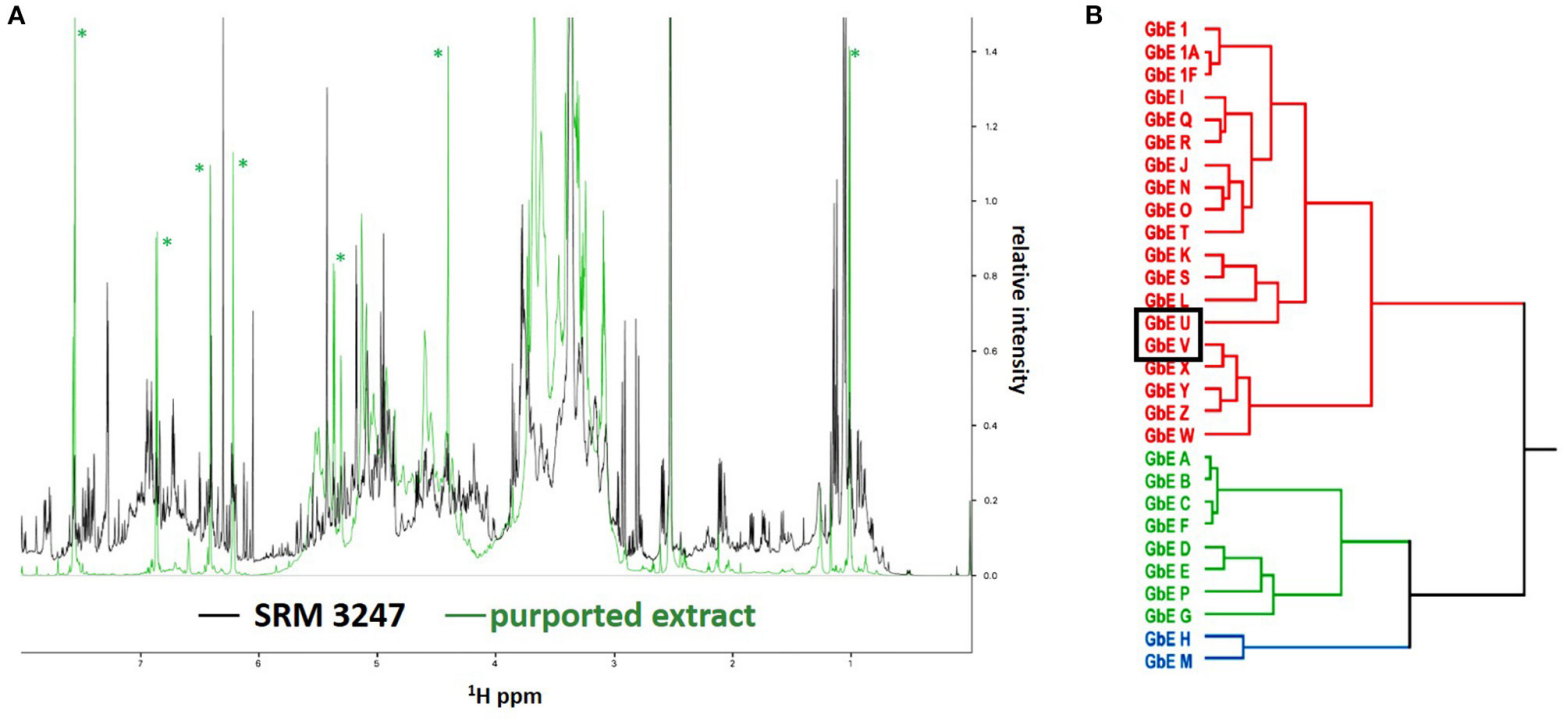
**(A)** Overlay of NMR spectra analyses of NIST SRM 3247 Ginkgo (*G. biloba* L.) extract (black trace) vs. a product obtained from the market purported to be Ginkgo extract (green line). Differences in peak frequencies and intensities were noted in the aromatic and aliphatic regions (6–8 and 1–3 ppm, respectively); green asterisks indicate rutin peaks. **(B)** Dendrogram analysis of the hierarchical clustering resulting from unsupervised analyses of NMR spectra of various Ginkgo products. Non-targeted NMR profiling of Ginkgo extracts (GbE) was conducted on NTP test articles (1, 1A, 1F), products procured from the U.S. market claimed as containing Ginkgo (A-T), NIST SRM 3247 Ginkgo extract (U), and NIST SRM 3248 Ginkgo SODF (V). Samples with spectra characteristic of Ginkgo clustered together (red), including NIST SRMs (black box), while samples with very low levels of flavonols and terpene trilactones were clustered separately (green and blue). Adapted with permission from Collins et al. (116), under Creative Commons license (https://creativecommons.org/licenses/by/4.0/legalcode).

Beyond the presence of known toxic constituents, questions of safety for a complex natural product may arise, such as whether consumption of a particular botanical species or extract thereof has long-term deleterious effects. Matrix-based CRMs have been leveraged in safety investigations in evaluating the identity and composition, and thus experimental relevance, of test articles. For example, safety studies conducted by the U.S. National Toxicology Program (NTP) attempt to identify potential harm of short- and long-term exposure to certain botanicals.^17^ NTP safety studies are designed to test botanical preparations that represent what the general public will be exposed to *via* the dietary supplement market. NTP researchers use authenticated reference materials in quantitative measurements of key bioactive or marker compounds as well as in qualitative non-targeted profiling to thoroughly determine the composition of candidate test articles to the extent practicable (115). This approach has been used following the NTP studies of *G. biloba* (Figure 7) (116–118), and in advance of studies on *A. racemose*—(119) and *Echinacea purpurea* (L.) Moench (119, 120). Importantly, these characterizations are reported openly, enabling improved research reproducibility and allowing a critical review of the composition of the specific test article chosen for each NTP study. In this way, the merits of botanical safety studies can be assessed in part based on how well the selected test article was representative of products on the market, authenticated reference materials (when available), and consensus quality standards (121).

Notably, these assessments of the chemical similarities and differences between reference materials, commercial ingredients/products, and experimental preparations by NTP researchers underscore how the inherent complexity and variability of NPs can confound research on their safety. For example, investigation of more than a dozen preparations of *E. purpurea* and *A. racemosa* demonstrated how samples could match characteristics of authentic material in orthogonal targeted and non-targeted chemical profile screening and yet yield different or even opposing bioactivities in CYP450 gene expression assays (119). Furthermore, the influence of sample preparation techniques is highlighted by the observation that hydrolyzing glycosides to the corresponding aglycones resulted in a decreased ability to chemically differentiate between authenticated and characteristic *G. biloba* materials and those samples that had been deemed as uncharacteristic (116). When certified reference materials are available, as was the case for *G. biloba* extract, their use can be vital to identifying and confidently addressing these analytical challenges.

## Discussion

As described in the above case studies, the use of reference materials and validated analytical methods in NP biomedical research promote accurate and reliable measurements of dietary constituents and their metabolites (Table 8). Unsurprisingly, the DS matrix CRMs with the highest prevalence of reported use are those that have been available for nearly a decade (i.e., multivitamin/multielement, fish and botanical oils, Ginkgo, and green tea). However, several important botanical matrix CRMs have been released in recent years or are near completion that significantly broaden the library of available materials. These CRMs, which are listed in Table 1, include kelp (*Thallus laminariae*), yerba mate (*Ilex paraguariensis* A.St.-Hil.) leaves, turmeric (*Curcuma longa* L.), ginger (*Z. officinale*), kudzu [*Pueraria montana* var. *lobata* (Willd.) Maesen and S.M. Almeida ex Sanjappa and Predeep], and Asian ginseng (*P. ginseng*). These newly available CRMs should find extensive use in not only method development/validation and quality control applications but also in novel research applications. In addition, several more botanical-derived DS ingredients are currently under development by NIH-ODS/NIST as candidate matrix SRMs, including black cohosh (*A. racemosa*), yohimbe [*Pausinystalia johimbe* (K. Schum.) Pierre ex Beille], eleuthero [*Eleutherococcus senticosus* (Rupr. and Maxim.) Maxim.], and ashwagandha [*Withania somnifera* (L.) Dunal] (Table 3). Although still under development, in certain circumstances these candidate DS SRMs may be made available to researchers who are initiating studies involving the use and/or characterization of interventions derived from these botanicals^18^.

**Table 8:** Examples of reported uses of NIST botanical matrix SRMs.

**SRM**	**References**	**Uses**			**Comments**
		**Method dev./ valid**.	**QC**	**Novel research**	
**Ephedra**					
3243 3244	Andrews et al. (77)		X		SRMs analyzed as controls for the determination of caffeine
**Green tea**					
3254 3255 3256	Castro et al. (122)	X			Method development for quantification of caffeine and catechins using LC-particle beam/electron ionization MS
3255	Napolitano et al. (102)	X			Compared qNMR and LC-MS/MS methods for quantification of catechins using SRM for assessment of accuracy
3254	Punyasiri et al. (80)	X			SRM used to validate a new sample preparation method involving freeze drying of the samples prior to extraction and analysis
3254 3255 3256	Andrews et al. (105)		X		Used as QC material to evaluate laboratory capabilities and measurement performance for catechins and caffeine
3254	Kellogg et al. (81)	X			Comparison of conventional and accelerated-solvent extraction for catechins from green tea; SRM used as one of four samples evaluated; not compared to certified values
3254 3255 3256	Kellogg et al. (83)			X	Comparison of metabolomic approaches for assessing variability of botanical green tea preparations including SRMs as reference samples
3254	Tian et al. (82)			X	SRM used in studies of intestinal UDP-glucuronosyltransferase inhibitors in green tea using a biochemometric approach
3254 3248	Crighton et al. (73)			X	Investigating application of DSA-TOF for screening adulterated dietary supplements
3254 3255 3256	Gusev et al. (106)		X		Used in a disintegration and dissolution testing study for green tea dietary supplements to evaluate formulation performance
3254 3255 3256	Clark et al. (84)			X	SRMs used as sample for interlaboratory comparison of untargeted MS to assess variability in metabolomic studies
***Ginkgo biloba***					
3246	Castro et al. (123)	X			Validation of sample preparation and detection of elements by ICP-OES
3246 3248	Booker et al. (101)	X			SRMs included in study of adulteration of *Ginkgo biloba* products; HPTLC analysis shown in paper includes SRMs
3247 3248	Catlin et al. (117)			X	SRMs used to determine chemical and biological similarity of *Ginkgo biloba* extracts
3247	Collins et al. (116)			X	SRMs used in non-targeted and targeted chromatographic and spectrophotometric studies of 24 commercially available *Ginkgo biloba* extracts
**Saw palmetto**					
3251	Srigley et al. (97)		X		Used in the analysis of virgin olive oil for determination of desmethlsterols, campesterol, stigmasterol, and β-sitosterol.
3251	Penugonda and Lindshield (96)		X		Used for the determination of fatty acids and phytosterols in commercial saw palmetto supplements
**Botanical oils**					
3274	Ahn et al. (93)	X			Validation of GC-MS method for fatty acids in food supplement oil products; comparison with certified values
**Fish oils**					
3275	Khoomrung et al. (92)	X			Validation of sample preparation method for fatty acid methyl esters using microwave-assisted derivation
3275	Srigley and Rader (90)	X			Determination of fatty acids in various fish oil supplements; chromatograms for analysis of SRM 3275 and comparison to certified values using a novel ionic liquid stationary phase for method validation
3275	Weatherly et al. (91)	X			Evaluated ionic liquid GC phases for separation of fatty acids; compared results for three fatty acids
3275	Karunathilaka et al. (86)	X			Validation of portable FTIR Device for prediction of fatty acid content in marine oil omega-3 dietary supplements
3275	Karunathilaka et al. (88)	X			SRMs used to validate ATR-FTIR and FT-NIR chemometric method for quantification of fatty acids
3275	Trbovic et al. (124)		X		Used for QC in GC-FID method for fatty acids in fish tissue and feed
3275	Li and Srigley (87)	X			Validation of GC-FID method for log chain omega-3 polyunsaturated fatty acids in chewable gel dietary supplements
**Soy**					
3238	Zhang et al. (125)	X			Development and validation of LC-particle beam/electron ionization MS for determination of isoflavones
3234	Kambhampati et al. (126)			X	Method development for protein quantification *via* determination of amino acids
**Turmeric**					
3299 3300	Mudge et al. (111)	X			Used in multi-laboratory study for determination of curcuminoids in turmeric dietary supplements by HPLC-DAD

*Uses include method development and/or validation, quality control (QC), and novel research investigations*.

## Conclusion

Scientists conducting NP research are encouraged to utilize the approaches for analytical method validation and the growing number of RM resources described herein to improve the overall quality of chemical measurements and to expand the knowledge base on the chemical characterization of NPs and DS. The enhanced analytical capacity that comes from leveraging reference materials and validated methods, in turn, optimizes the evidence base for dietary guidelines and healthcare practice as it relates to the use of dietary interventions to help maintain health and reduce illness.

## Author Contributions

All authors jointly conceived, developed, drafted, and edited this manuscript.

## Funding

This work was supported by the NIH Office of Dietary Supplements, as an activity of the Analytical Methods and Reference Materials Program (https://ods.od.nih.gov/Research/AMRMProgramWebsite.aspx).

## Conflict of Interest

The authors declare that the research was conducted in the absence of any commercial or financial relationships that could be construed as a potential conflict of interest.

## Publisher's Note

All claims expressed in this article are solely those of the authors and do not necessarily represent those of their affiliated organizations, or those of the publisher, the editors and the reviewers. Any product that may be evaluated in this article, or claim that may be made by its manufacturer, is not guaranteed or endorsed by the publisher.

## Footnotes

^1^ISO/Guide 30:2015 - Reference Materials: https://www.iso.org/obp/ui/#iso:std:iso:guide:30:ed-3:v1:en.

^2^AOAC International Official Methods of Analysis, Appendix K: http://www.eoma.aoac.org/app_k.pdf.

^3^FDA Bioanalytical Method Validation Guidance for Industry: https://www.fda.gov/media/70858/download.

^4^FDA Guidelines for the Validation of Analytical Methods for Nucleic Acid Sequence-Based Analysis of Food, Feed, Cosmetics and Veterinary Products: https://www.fda.gov/media/121751/download.

^5^NIH-ODS Analytical Methods and Reference Materials Program: https://ods.od.nih.gov/Research/AMRMProgramWebsite.aspx.

^6^NIST Measurements and Standards for Botanical Dietary Supplements: https://www.nist.gov/programs-projects/measurements-and-standards-botanical-dietary-supplements.

^7^Cerilliant Corp Analytical Reference Standards: https://www.cerilliant.com/products/catalog.aspx.

^8^AHP-Verified Botanical Reference Materials: https://herbal-ahp.org/botanical-reference-materials/.

^9^ChromaDex Reference Standards: https://www.chromadex.com/standards-overview/.

^10^PhytoLab Reference Substances: https://www.phytolab.com/en/our-services/reference-substances-phyproof/.

^11^Alkemist Labs Reference Materials: https://www.alkemist.com/reference-materials/.

^12^Extrasynthese Botanical Materials and Quantified Reference Extracts: https://www.extrasynthese.com/.

^13^NRC Canada CRMs: https://nrc.canada.ca/en/certifications-evaluations-standards/certified-reference-materials.

^14^NIST DSQAP: https://www.nist.gov/programs-projects/dietary-supplement-laboratory-quality-assurance-program.

^15^NIST HAMQAP: https://www.nist.gov/programs-projects/health-assessment-measurements-quality-assurance-program.

^16^NIST Interagency/Internal Reports: https://www.nist.gov/nist-pub-series/nist-interagencyinternal-report-nistir.

^17^NTP Botanical Dietary Supplements: https://ntp.niehs.nih.gov/whatwestudy/topics/botanical/index.html.

^18^NIH-ODS AMRM Program RMs for DS Analysis: https://ods.od.nih.gov/Research/AMRMReferenceMaterials.asp
